# The H3K27M mutation alters stem cell growth, epigenetic regulation, and differentiation potential

**DOI:** 10.1186/s12915-022-01324-0

**Published:** 2022-05-30

**Authors:** N. Kfoury-Beaumont, R. Prakasam, S. Pondugula, J. S. Lagas, S. Matkovich, P. Gontarz, L. Yang, H. Yano, A. H. Kim, J. B. Rubin, K. L. Kroll

**Affiliations:** 1 Department of Neurosurgery, University of California in San Diego, La Jolla, CA USA; 2 Department of Developmental Biology, Washington University School of Medicine, St Louis, MO USA; 3 Department of Pediatrics, Washington University School of Medicine, St Louis, MO USA; 4 Center for Cardiovascular Research, Department of Medicine, Washington University School of Medicine, St Louis, MO USA; 5 Department of Neurological Surgery, Washington University School of Medicine, St Louis, MO USA; 6 The Brain Tumor Center, Washington University School of Medicine, Siteman Cancer Center, St. Louis, MO USA; 7 Department of Neuroscience, Washington University School of Medicine, St Louis, MO USA

**Keywords:** DIPG, H3K27M, Epigenetics, Aberrant differentiation, Pediatric brain tumors

## Abstract

**Background:**

Neurodevelopmental disorders increase brain tumor risk, suggesting that normal brain development may have protective properties. Mutations in epigenetic regulators are common in pediatric brain tumors, highlighting a potentially central role for disrupted epigenetic regulation of normal brain development in tumorigenesis. For example, lysine 27 to methionine mutation (H3K27M) in the *H3F3A* gene occurs frequently in Diffuse Intrinsic Pontine Gliomas (DIPGs), the most aggressive pediatric glioma. As H3K27M mutation is necessary but insufficient to cause DIPGs, it is accompanied by additional mutations in tumors. However, how H3K27M alone increases vulnerability to DIPG tumorigenesis remains unclear.

**Results:**

Here, we used human embryonic stem cell models with this mutation, in the absence of other DIPG contributory mutations, to investigate how H3K27M alters cellular proliferation and differentiation. We found that H3K27M increased stem cell proliferation and stem cell properties. It interfered with differentiation, promoting anomalous mesodermal and ectodermal gene expression during both multi-lineage and germ layer-specific cell specification, and blocking normal differentiation into neuroectoderm. H3K27M mutant clones exhibited transcriptomic diversity relative to the more homogeneous wildtype population, suggesting reduced fidelity of gene regulation, with aberrant expression of genes involved in stem cell regulation, differentiation, and tumorigenesis. These phenomena were associated with global loss of H3K27me3 and concordant loss of DNA methylation at specific genes in H3K27M-expressing cells.

**Conclusions:**

Together, these data suggest that H3K27M mutation disrupts normal differentiation, maintaining a partially differentiated state with elevated clonogenicity during early development. This disrupted response to early developmental cues could promote tissue properties that enable acquisition of additional mutations that cooperate with H3K27M mutation in genesis of DMG/DIPG. Therefore, this work demonstrates for the first time that H3K27M mutation confers vulnerability to gliomagenesis through persistent clonogenicity and aberrant differentiation and defines associated alterations of histone and DNA methylation.

**Supplementary Information:**

The online version contains supplementary material available at 10.1186/s12915-022-01324-0.

## Background

Pluripotent stem cells differentiate to form every complex tissue structure of multicellular life. The progression of pluripotent stem cells to a differentiated cell state is mediated by epigenetic modification of DNA and histone proteins, including methylation or acetylation of the lysine 27 position on histone H3 (H3K27). H3K27 trimethylation (H3K27me3) modifications of chromatin increase during differentiation, resulting in wide-scale gene repression and heterochromatization [1, 2]. Concordantly, differentiated cells lose their self-renewal capacity and their pluripotency. This is essential for normal cellular function and as a protection against cancer. However, in some cancers, driver mutations in key aspects of epigenetic regulation disrupt normal differentiation and result in persistence of aberrant clonogenicity and multi-potency. Cells that are affected are at a heightened risk for malignant transformation.

One critical example of this may be the mechanisms by which lysine 27 to methionine mutation of histone H3 drives the genesis of Diffuse Midline Gliomas (DMG), including Diffuse Intrinsic Pontine Gliomas (DIPGs). DIPGs are the leading cause of brain tumor-associated mortality in the pediatric population [3]. They exhibit a unique neurodevelopmental pattern of occurrence, affecting only the pons, and only early in life [3]. H3K27M interferes with the Polycomb repressive complex 2 (PRC2), which deposits H3K27me3 modifications through an, as of yet, unidentified molecular mechanism [4, 5]. Not only does this interference prevent the addition of trimethylation marks at mutant histones but it also prevents “spreading” of H3K27me3 to wild-type histones throughout the genome [4, 5]. This results in a global loss of H3K27me3, a consequent increase in H3K27 acetylation (H3K27ac), and aberrant gene activation [6, 7]. Of note, H3K27me3 is preferentially retained at unmethylated CpG islands, which also happen to be strong PRC2 targets, subsequently inhibiting the expression of crucial tumor suppressor genes [8].

While it is known how H3K27M affects the epigenetic state of mutant cells, it is currently unknown how this altered epigenetic state contributes to tumorigenesis. H3K27M mutation is necessary but not sufficient to cause cancer, and several studies have shown links between this histone mutation and changes in expression of genes that regulate neural differentiation [7, 9, 10]. Therefore, the growing consensus in the field is that H3K27M primes cells for tumorigenesis by altering their global epigenetic state, potentially interfering with normal differentiation, and stalling mutant cells at a stem cell stage of development [11, 12]. However, what the H3K27M mutation does in isolation to alter developmental potential or stem cell state is not well established and has not been directly demonstrated. Therefore, utilizing a developmental system to understand this mutation and its effects on differentiation in isolation could prove to be valuable in uncovering some of the specific mechanisms that may underlie tumorigenesis.

Here, we utilize pairs of isogenic human embryonic stem cell lines with or without the H3K27M mutation to investigate mechanisms by which H3K27M mutation drives tumorigenesis. We assessed the effect of H3K27M on multiple stem cell differentiation paradigms, including trilineage, ecto-, endo-, and mesodermal differentiation, and neural differentiation. This revealed some common features of gene dysregulation that occur in cells with H3K27M mutation. We further linked a failure of normal neural specification and differentiation to alterations of histone and DNA methylation. In this work, H3K27M increased stem cell proliferation and stem cell properties and interfered with differentiation, resulting in loss of most H3K27me3 marks and in anomalous onset of expression of developmental genes during multilineage or directed differentiation. These data provide key support for the hypothesis that the H3K27M mutation confers vulnerability to gliomagenesis through persistent clonogenicity and aberrant differentiation.

## Results

### The H3K27M mutation confers higher growth potential and clonogenicity in human stem cells

The H3K27M mutation is necessary but not sufficient to drive DIPG tumorigenesis. Instead, it may confer a cellular context in which additional mutations can do so. H3K27M mutations can occur in one of two histone H3 variants, either H3.1 or H3.3 (written as H3.1K27M and H3.3K27M). The prevalence of H3.1K27M is ~20% in histone H3 mutant DMG cases whereas, H3.3K27M is ~80% [9, 13–17]. H3.3K27M mutation was chosen for this study as it is the predominant histone H3 mutation found in DMG. To assess how this mutation could alter stem cell state and differentiation potential in the absence of other mutations, we generated a human embryonic stem cell (hESC) line with heterozygous knock-in of a single base mutation (A>T) in the *H3F3A* gene, resulting in a K to M amino acid substitution at position 27 in the protein (H3K27M). Multiple clones were derived and the A>T mutation was confirmed by Sanger sequencing (Additional file 1: Fig. S1A-B). In addition, each clone was determined to have a normal karyotype (Additional file 1: Fig. S1C). Five clones were used in further experimental work (Additional file 2: Table S1). All clones were found to express the H3K27M mutant protein by western blot analysis (Additional file 1: Fig. S1D-E).

**Fig. 1:**
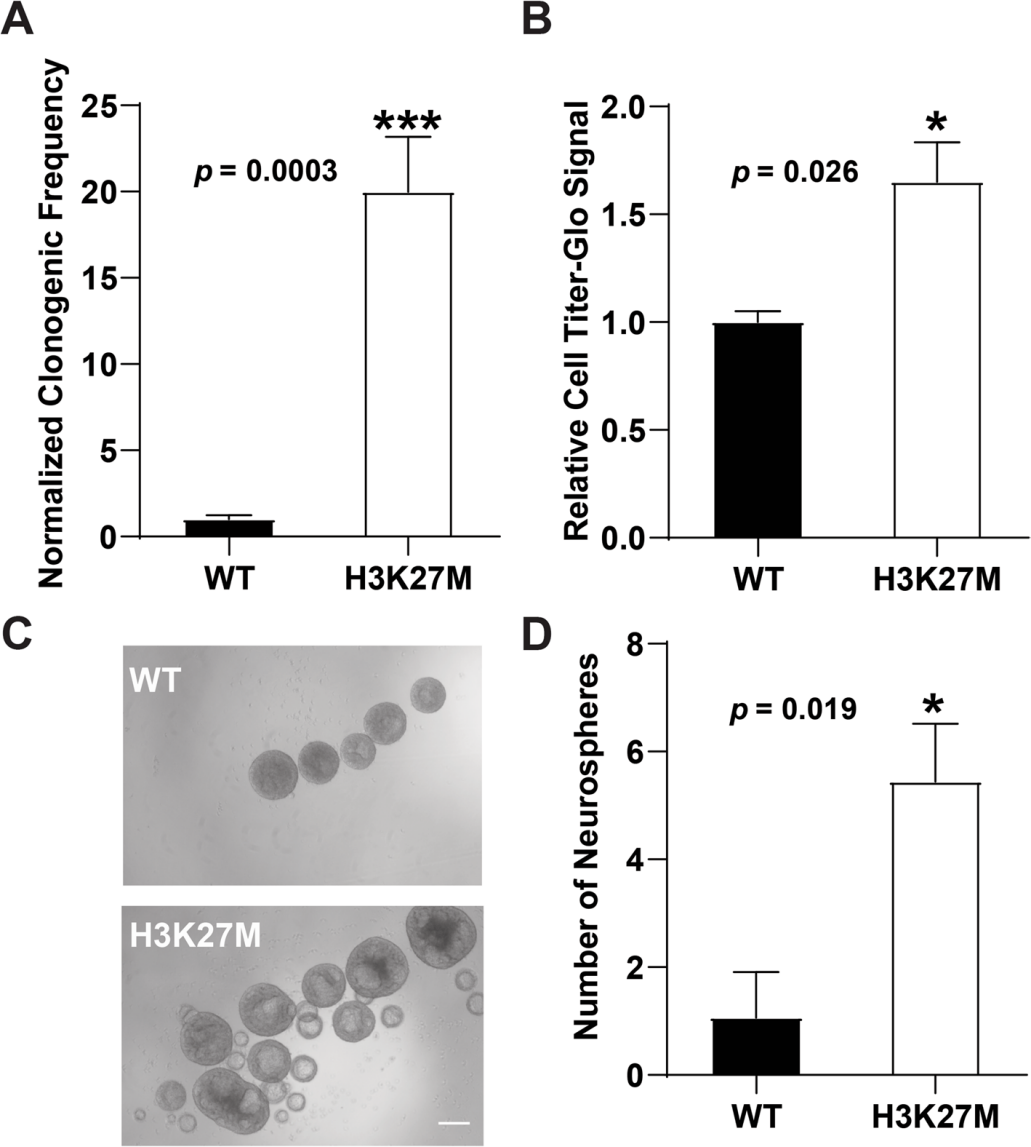
Human embryonic stem cells with H3K27M mutation have increased clonogenic stem cell properties. **A** Percentages of stem cells with clonogenic potential were assessed by plating on low attachment plates to promote sphere formation at extreme limiting dilution (ELDA). Data shown is an average +/− sem of four biological replicates utilizing four H3K27M clonal lines, each performed in technical triplicate. **B** hESCs were grown as 3-D spheres in maintenance media for 72 h and cell number was assessed by using the CellTiter-Glo assay (Promega, see Methods). Clones used and biological replicates performed are in Additional file 2: Table S1, with each biological replicate performed in technical triplicate, and WT values set at 1.0 for comparison with the K27M sample. **C** Representative bright field image of WT and H3K27M hESCs after 72 h of growth in non-adherent culture in maintenance media (scale bar=100μm). **D** 120 hESCs were plated under non-adherent conditions in maintenance media and the number of spheres that formed was assessed at day 7. Clones used for each biological replicate experiment and numbers of biological replicate experiments performed are described in Additional file 2: Table S1 for each finding in this manuscript. *p* values were calculated using an unpaired *t*-test

We first assessed whether introduction of the H3K27M mutation altered clonogenic cell activity using an extreme limiting dilution assay (ELDA; Fig. 1A). Cells were seeded onto low attachment plates in hESC maintenance media and sphere formation over 7 days was used to define clonogenic potential. By comparison with wild type (WT) hESCs, lines carrying the H3K27M mutation had a significantly elevated capacity for clonogenic growth (Fig. 1A and Additional file 2: Table S1). We next determined whether increased clonogenicity in K27M-expressing cells was associated with increased cell number over a 72-hr. period using the Cell Titer-Glo assay. By comparison with WT hESCs, H3K27M hESC spheres exhibited increased cell number (Fig. 1B and Additional file 2: Table S1) and concordantly, were also of a larger size (Fig. 1C). In addition, we observed that H3K27M cells formed a greater number of spheres than WT hESCs (Fig 1D and Additional file 2: Table S1).

### H3K27M mutation alters the differentiation potential of hESCs

**Fig. 2:**
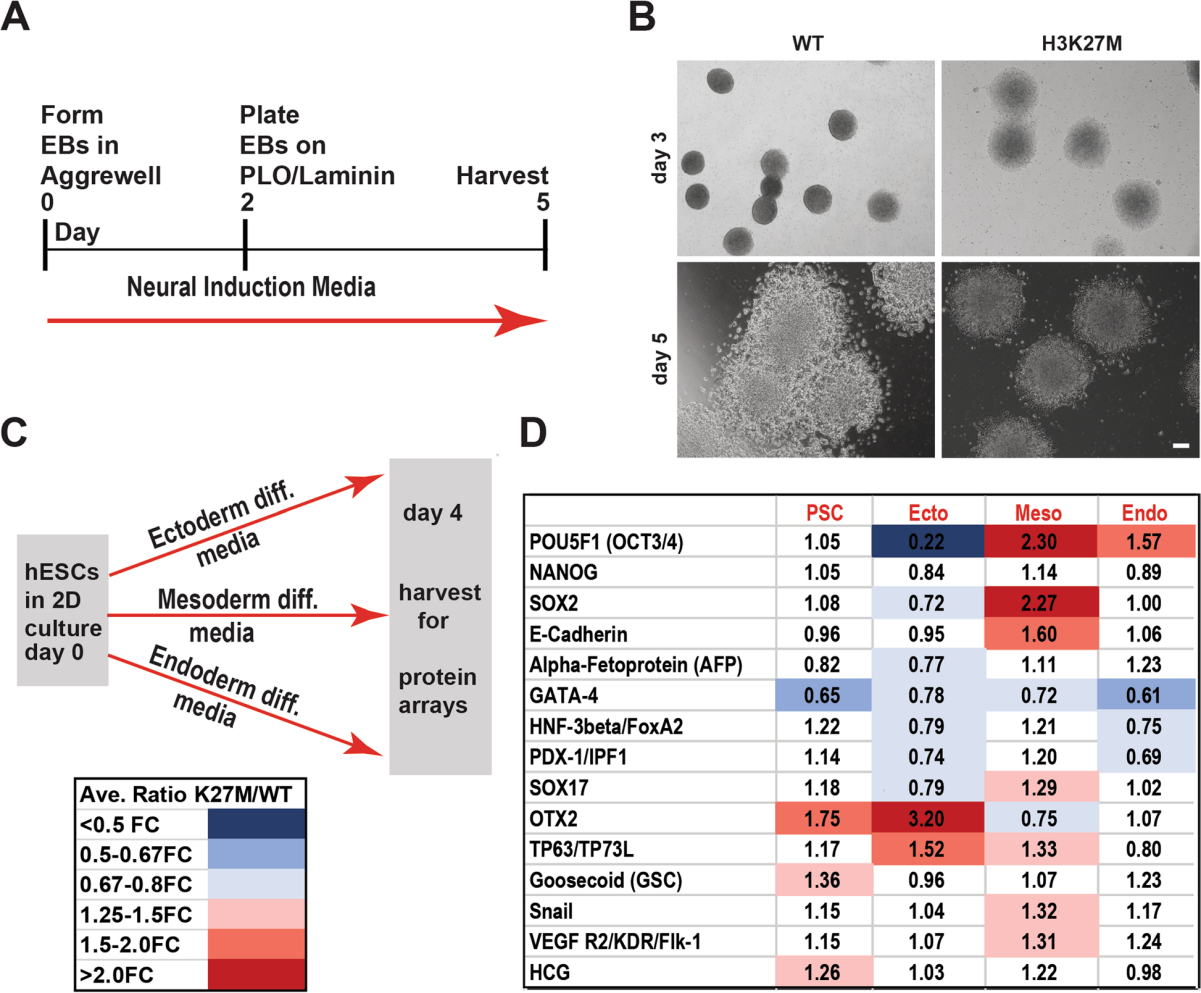
Altered tri-lineage differentiation of hESCs carrying H3K27M mutation. **A** Schematic of the neural induction scheme used (see the “Methods” section for details). **B** Representative bright field images of plated EBs at 3 and 5 days after initiating differentiation with neural induction media (scale bar=100μm). **C** Schematic of the tri-lineage differentiation scheme utilized for protein array analysis (see the “Methods” section). **D** Analysis of tri-lineage marker expression by protein array. Summary of fold differences between H3K27M and WT hESCs, when maintained in hESC/pluripotent stem cell (PSC) maintenance media or induced to undergo ectodermal, mesodermal, or endodermal differentiation. After normalization of intensity values on each array with internal controls, fold differences between the expression of each gene in the H3K27M versus the WT hESC line under each differentiation condition were calculated and averaged across arrays performed with six independent replicate sample sets utilizing two clonal lines. Clones used for each biological replicate experiment and numbers of biological replicate experiments performed are described in Additional file 2: Table S1

Failure to normally differentiate increases the vulnerability of cells for transformation and therefore, we sought to determine whether H3K27M altered differentiation. hESCs were induced to undergo specification as cortical neural progenitor cells by culture as embryoid bodies (EBs) in neural induction media for two days, followed by plating on poly-L-ornithine (PLO)/Laminin for 3 days as described (Fig. 2A; “Methods”). Consistent with the results above, EBs derived from hESCs carrying the H3K27M mutation were larger than WT EBs at day 3. By day 5, WT EBs exhibited morphological features consistent with specification as neural progenitors, including the formation of neural rosette structures. These overt signs of neural progenitor specification were compromised in EBs with the H3K27M mutation (Fig. 2B).

To assess whether the H3K27M mutation might also alter the capacity for hESCs to be specified as a range of other cell types, we evaluated the capacity of the hESCs to differentiate into the three germ layers by specification as ectoderm, mesoderm, or endoderm in defined media, as described in the “Methods” section (Fig. 2C). Four days after initiating each differentiation scheme, protein samples were collected and subjected to a protein array to analyze the relative expression of 15 protein markers, including markers of hESCs and of the three germ layers. Normalized fold differences in marker expression between the H3K27M and WT samples were assessed. When the cells were maintained in hESC media, most markers were not differentially expressed between the H3K27M and WT models; however, OTX2, GSC, and HCG, markers of ectoderm, mesoderm, and trophectoderm, respectively, were all elevated in the H3K27M line. Greater differences in gene expression were observed upon differentiation, particularly into mesoderm: here markers of undifferentiated hESCs (POU5F1, SOX2) and undifferentiated epithelium (E-Cadherin) remained elevated in the H3K27M cells (Fig. 2D and Additional file 2: Table S1) while markers of mesoderm (Flk-1 and Snail, which are also associated with cells undergoing epithelial to mesenchymal transition) and of other germ layers (e.g., SOX17, TP63) were also elevated. Likewise, in the ectodermal differentiation scheme, the capacity to express OTX2 (an ectodermal germ layer and mesenchymal marker) and TP63 (an epidermal marker) was increased (Fig. 2D and Additional file 2: Table S1). Together, these differences in marker expression suggested that the H3K27M mutation may compromise the capacity of hESCs to down-regulate stem cell and epithelial markers while also deregulating some aspects of germ layer specification-related gene expression. This latter property could lead to increased capacity of H3K27M cells to respond to ectodermal and mesodermal developmental patterning cues by activating some markers of those germ layers (Fig. 2D). Together, these data suggest that H3K27M-expressing hESCs exhibit aberrant directed differentiation.

**Fig. 3:**
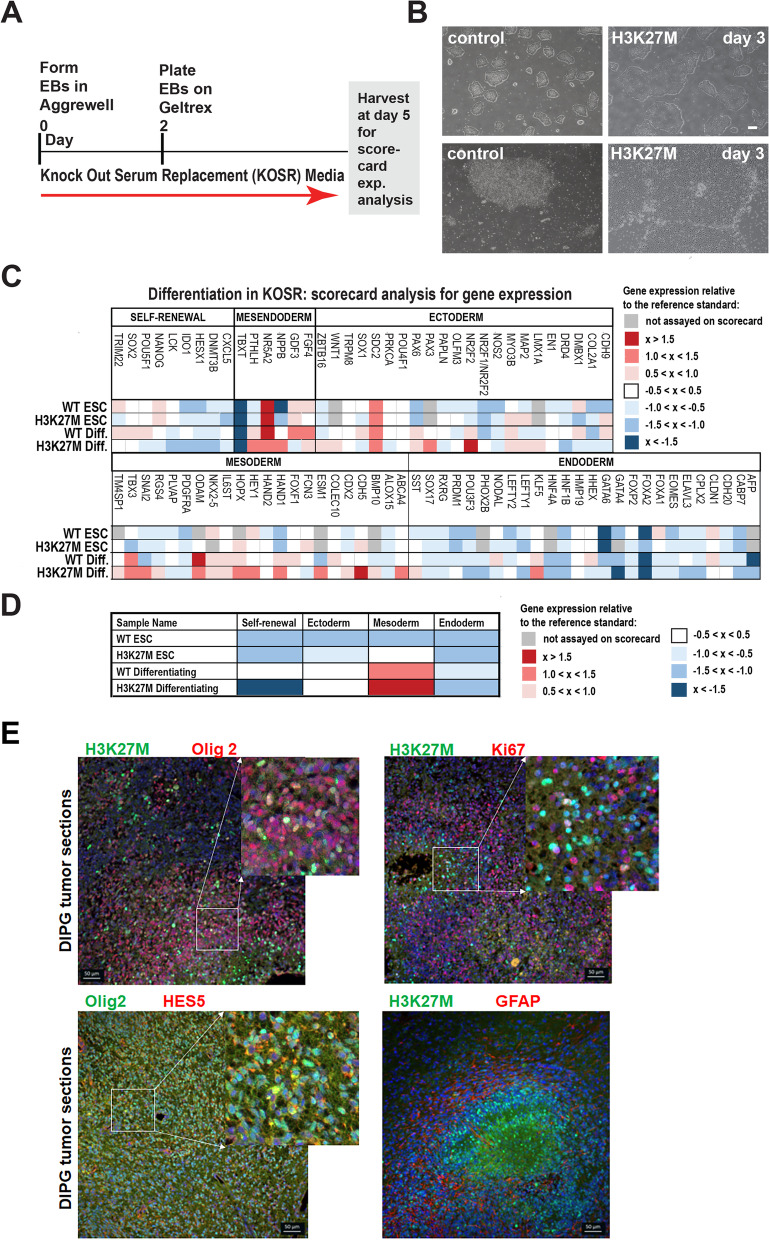
H3K27M hESCs undergoing undirected differentiation exhibit altered growth and increased expression of mesodermal and ectodermal genes. **A** Scheme for undirected differentiation in KOSR media and Scorecard analysis. **B** Representative bright field images of the WT and H3K27M models at day 3 of differentiation in KOSR (scale bar=100μm). **C**, **D** hESC Scorecard assays were performed on six independent biological replicate samples encompassing three different clonal lines carrying the H3K27M mutation (Additional file 2: Table S1), with gene expression fold changes relative to the reference standard indicated for each marker indicated in **C** and general expression trends for each category of markers shown in **D**. **E** Representative immunostaining images for the indicated proteins in RCAS DMG tumor tissue sections are shown. Scale bar=50μm

To further assess how the H3K27M mutation may have altered regulation of gene expression and cell fate acquisition, we allowed undirected cell specification to occur in KnockOut Serum Replacement (KOSR) media, as shown, providing conditions that were generally permissive for differentiation into the three germ layers (Fig. 3A). In bright field images of these cells after dissociation and plating (on day 3), H3K27M colonies were larger and had a more irregular appearance than those derived from the WT models (Fig. 3B). Cells from these models were then harvested either as ESCs (day 0) or at 5 days of specification and hESC Scorecard Assays were used to assess the relative expression of panels of marker genes for self-renewal, mesendoderm, mesoderm, endoderm, and ectoderm (see the “Methods” section).

In comparisons between WT and H3K27M mutant hESCs grown in maintenance media, the models exhibited only modest differences in marker gene expression (Fig. 3C, D and Additional file 2: Table S1). By contrast, upon differentiation in KOSR, the H3K27M line exhibited strongly reduced expression of self-renewal markers and increased expression of some ectodermal and many mesodermal markers (Fig. 3C, D and Additional file 2: Table S1). These included PAX3 and NR2F2 (ectoderm) and SNAI2, HOPX, HEY1, HAND1, ESM1, CDH5, and ABCA4 (mesoderm). To gain insight into whether the H3K27M mutation was also associated with undifferentiated cells in vivo, we took advantage of an established H3.3K27M RCAS mouse model of diffuse midline glioma [18]. In the tumors, H3K27M colocalized with Olig2, Hes5, and Ki67, markers of proliferating progenitor cells. By contrast, the mutation did not colocalize with GFAP or NeuN, markers of astrocytic and neuronal differentiation, respectively (Fig. 3E and Additional file 3: Fig. S2). Therefore, in vivo findings are concordant with the findings in our in vitro model.

### H3K27M mutation alters gene expression during neural cell specification

**Fig. 4:**
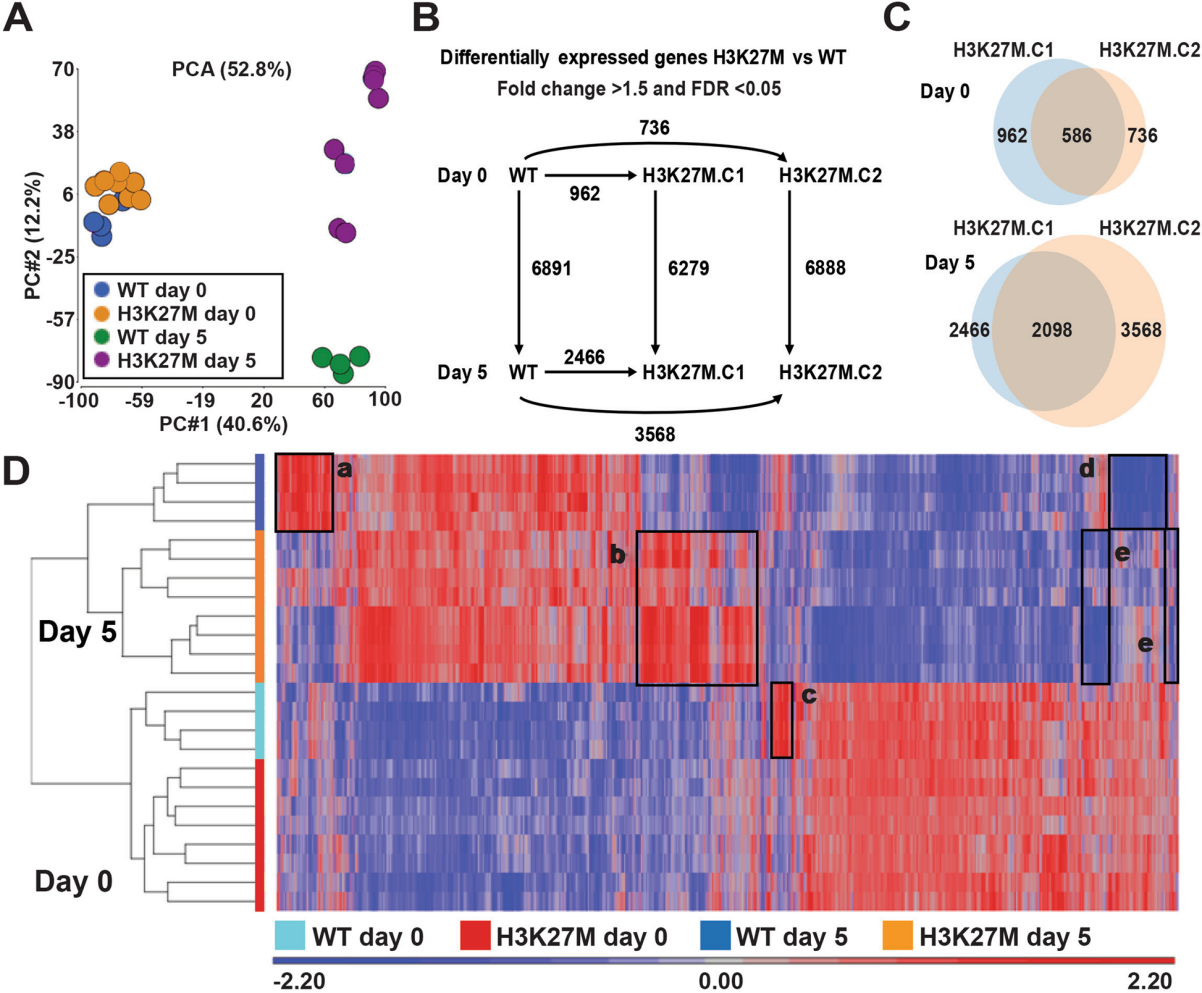
H3K27M mutation alters gene expression during neural cell specification. **A** Principal component analysis plot for RNA-seq datasets, corresponding to 8 H3K27M and 4 WT biological replicate samples for each time point (day 0, day 5). **B** Numbers of differentially expressed genes (DEGs) in comparisons between WT and H3K27M mutant clones 1 and 2, at day 0 and day 5. **C** Venn diagram shows the shared and H3K27M mutant clone specific DEGs at day 0 and day 5. **D** Unsupervised hierarchical clustering defined 5 clusters of genes (clusters a–e) that exhibit differential expression in WT versus H3K27M clones

As the H3K27M mutation had broad effects on trilineage differentiation, we next wondered whether it would also affect neural cell specification and differentiation. In our work above, we observed that, when hESCs with the H3K27M mutation were induced to form neural tissue, they formed larger EBs, while their differentiation and morphological organization into rosette structures appeared to be compromised (Fig. 2A, B). Therefore, to gain deeper insight into the mechanisms underlying this aberrant neural differentiation, we performed RNA sequencing of the WT and H3K27M mutant models, at day 0 (as hESCs) and at day 5 of neural cell specification. Substantially greater numbers of differentially expressed genes (DEGs) were obtained at day 5 than at day 0, indicating that the H3K27M mutation more readily disrupted gene expression during differentiation, versus when cells were grown in pluripotency maintenance media (Fig. 4A–C) [19]. Principal component analysis (PCA) of the gene expression data indicated that all WT and H3K27M clones clustered together at day 0. However, by day 5, while the WT clones clustered together and were uniformly different from day 0 WT cells, the H3K27M clones populated three different cell clusters, all distinct from day 0 WT and H3K27M cells, day 5 WT cells, and, from each other (Fig. 4A). The numbers of genes that exhibited differential expression between day 0 and day 5 were similar in the WT and the two H3K27M clones (Fig. 4B). Interestingly, on day 5, as indicated by the PCA plot, the two mutant clones exhibited substantial transcriptomic differences (Fig. 4C). These data confirmed that H3K27M-expressing hESCs exhibit aberrant differentiation and further underscore why their directed differentiation yields diverse cell phenotypes.

The differences between WT and H3K27M-expressing hESC clones on day 0 and day 5 were further visualized through unsupervised hierarchical clustering (Fig. 4D). Several important features for additional study were evident in the heatmap. Day 0 WT and H3K27M samples clustered together, separately from day 5 WT and H3K27M, which also clustered separately. Specific genes that distinguished day 5 WT versus H3K27M clones were identified, as were genes that distinguished the three distinct clusters of day 5 H3K27M cells (Fig. 4D, clusters a–b, d–e, and Fig. 4A, D three H3K27M profiles in cluster b; Additional file 4: Table S2). Finally, one set of genes distinguished the day 0 WT versus H3K27M cells (Fig. 4D, cluster c; Additional file 4: Table S2). Since the most pronounced trend was increased expression in the H3K27M cells at day 5 (cluster b), we validated a number of these DEGs by RT-qPCR, demonstrating upregulation of transcripts in the H3K27M line consistent with the findings from the RNA-seq analysis (Additional file 5: Fig. S3). Together, these data indicate that H3K27M mutation results in loss of high-fidelity regulation of gene expression as differentiation is induced, resulting in greater variability in gene expression. In the context of DIPG, a similar phenomenon could confer increased vulnerability to transformation.

**Fig. 5:**
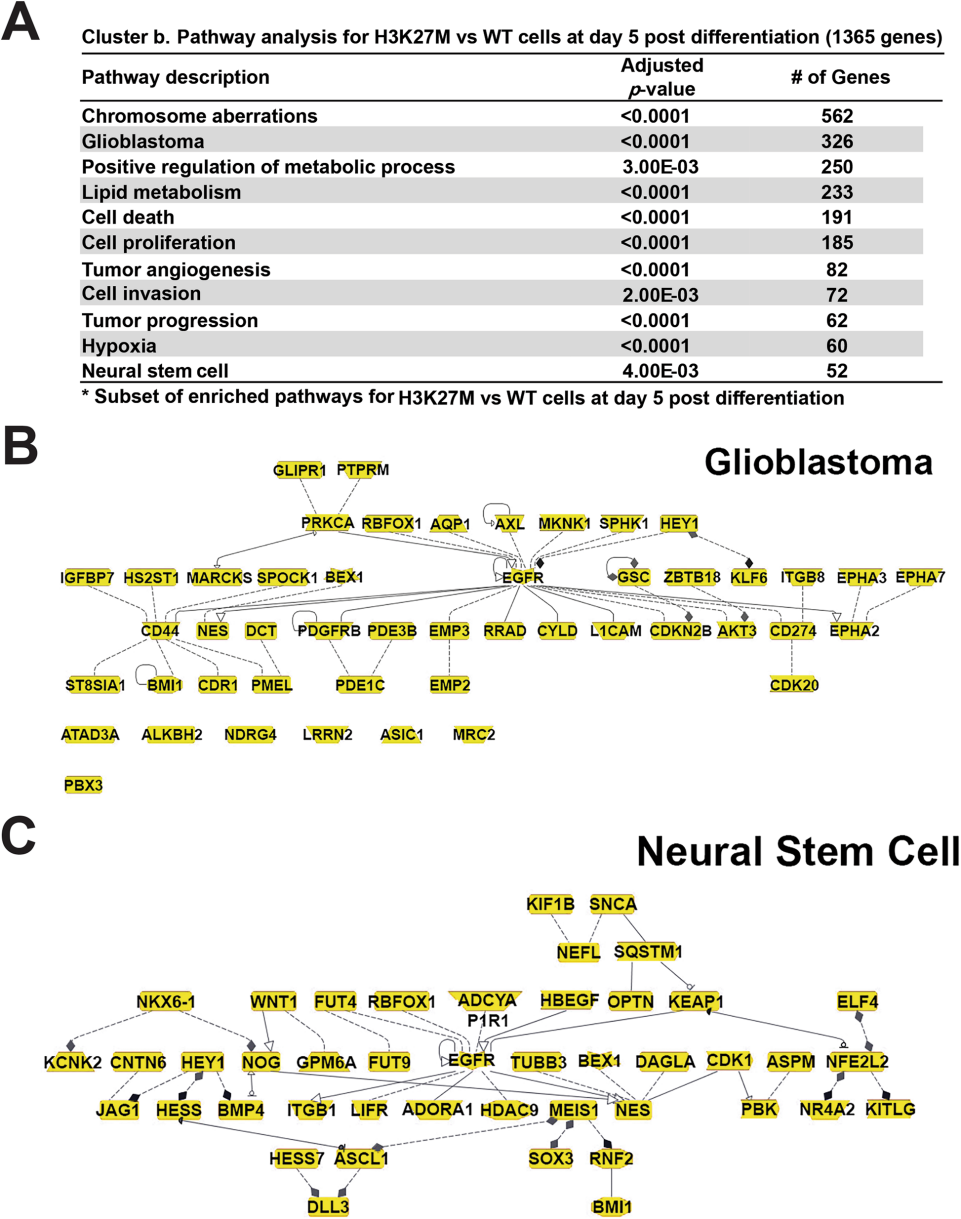
Enriched pathways and gene networks with elevated expression in the H3K27M cells vs WT, at day 5. Analysis of differentially expressed genes in Figure 4 cluster b, which showed elevated expression in the mutant cells at day 5, revealed **A** enriched gene ontology terms, and **B** and **C** enriched networks of genes related to glioblastoma and neural stem cells

To further define the features of the genes that exhibited differential expression in the WT versus mutant lines (Fig. 4D, clusters a–e), we performed pathway analysis (Additional file 6: Table S3). Cluster b, which exhibited increased expression in H3K27M versus WT clones at day 5 was associated with many gene ontology (GO) terms related to cell proliferation and transformation, including a network of genes related to glioblastoma (Fig. 5A, B), and networks related to cell proliferation, tumor angiogenesis and progression, cell invasion, and neural stem cells (Fig. 5A–C, Additional file 7: Fig. S4). Cluster d, which exhibited decreased expression in WT versus H3K27M clones at day 5, was enriched both for genes related to regulation of cell proliferation/division and transformation and to cell differentiation and growth arrest (Additional file 6: Table S3). These data indicate that the presence of the H3K27M mutation perturbs the expression of genes that regulate cell proliferation and differentiation.

### H3K27M mutation causes widespread loss of H3K27me3 and a less pronounced reduction of DNA methylation

**Fig. 6:**
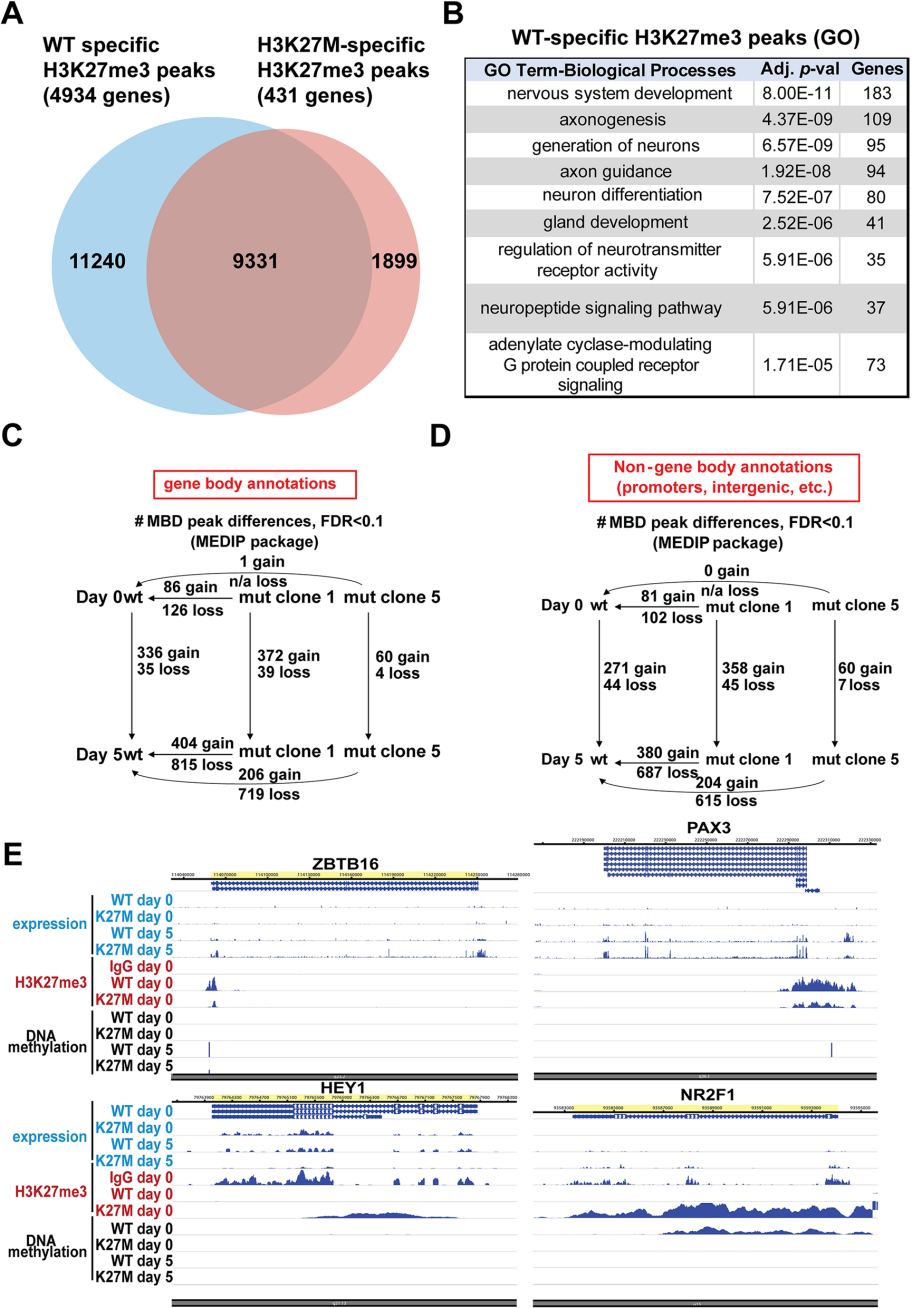
Widespread loss of H3K27me3 and reduced DNA methylation is seen in the H3K27M mutant line. **A** Venn diagram indicates the WT-specific, H3K27M mutant-specific, and shared peaks and genes in hESCs. **B** GO terms (biological processes) enriched in genes associated with the WT-specific H3K27me3. **C**, **D** Numbers of gene bodies (**C**) and non-gene body regions (**D**) with differential DNA methylation in comparisons of the WT and H3K27M mutant (clones 1 and 2) at day 0 and day 5. **E** Browser track views for several marker genes that exhibit the predominant trends

To understand the mechanisms that might underlie this disrupted gene expression, we analyzed how H3K27me3 was altered in the WT versus H3K27M hESCs. A striking disruption of H3K27me3 was seen, with WT stem cells exhibiting 11240 sites of H3K27me3 not found in mutant cells, while the mutant had only 1899 specific sites of H3K27me3 not seen in the WT cells (Fig. 6A, Additional file 8: Table S4) [19]. These differentially methylated regions correspond to 4934 genes associated with H3K27me3 in the WT and not mutant, and only 431 differentially methylated regions with H3K27me3 in the mutant and not the wildtype. This dramatic loss of H3K27me3 is already evident in the mutant hESCs, although it doesn't result in substantial alteration of gene expression when the cells are cultured in pluripotency maintenance media (Fig. 4). Gene ontology (GO) analysis of WT-specific H3K27me3 peaks indicated that these were related to genes involved in nervous system development, including many terms related to neuronal differentiation (e.g., axonogenesis, regulation of neurotransmitter receptor activity, neuron differentiation; Fig. 6B). These data suggest that the H3K27M mutation causes loss of the repressive H3K27me3 modification at a wide range of genes involved in neuronal differentiation, which could account for the aberrant expression of both neural and non-neural genes that we observed in the protein arrays.

While H3K27me3 restrains the expression of developmental genes in stem cells and dynamically controls their expression during cellular differentiation, DNA methylation is also important for repressing gene expression. Therefore, we also performed methyl-CpG binding domain sequencing (MBD-seq) to assess how DNA methylation was altered in H3K27M versus WT cells at day 0 and day 5. Relative to WT cells, H3K27M cells predominantly lost DNA methylation and this trend was seen predominantly in day 5 cells (Fig. 6C, D). However, as opposed to the changes in H3K27me3, where >11,000 differential peaks were lost in the H3K27M cells relative to WT, only hundreds of DNA methylation peaks were lost in the H3K27M cells relative to WT, in either gene bodies or non-gene bodies (e.g., promoters and intergenic regions; Fig. 6C, D, Additional file 9: Table S5). As was seen for H3K27me3 peaks that were lost in the mutant, DNA methylation peaks lost in the mutant were also highly enriched for genes involved in GO terms related to neural development at both day 0 and day 5, with top GO terms at day 0 including axonogenesis and axon guidance and top GO terms at day 5 including neuron differentiation and generation of neurons (Additional file 9: Table S5).

We examined browser views for marker genes that were dysregulated in the mutant cells in Figs. 2 and 3. These followed the trends above: most genes exhibited increased expression in mutant cells at day 5 but not day 0, while H3K27me3 peaks were dramatically reduced at these genes in the mutant hESCs (Fig. 6E). Most of these genes exhibited no differential DNA methylation, but a few showed reduced DNA methylation in the H3K27M cells at day 5 (Fig. 6E).

**Fig. 7:**
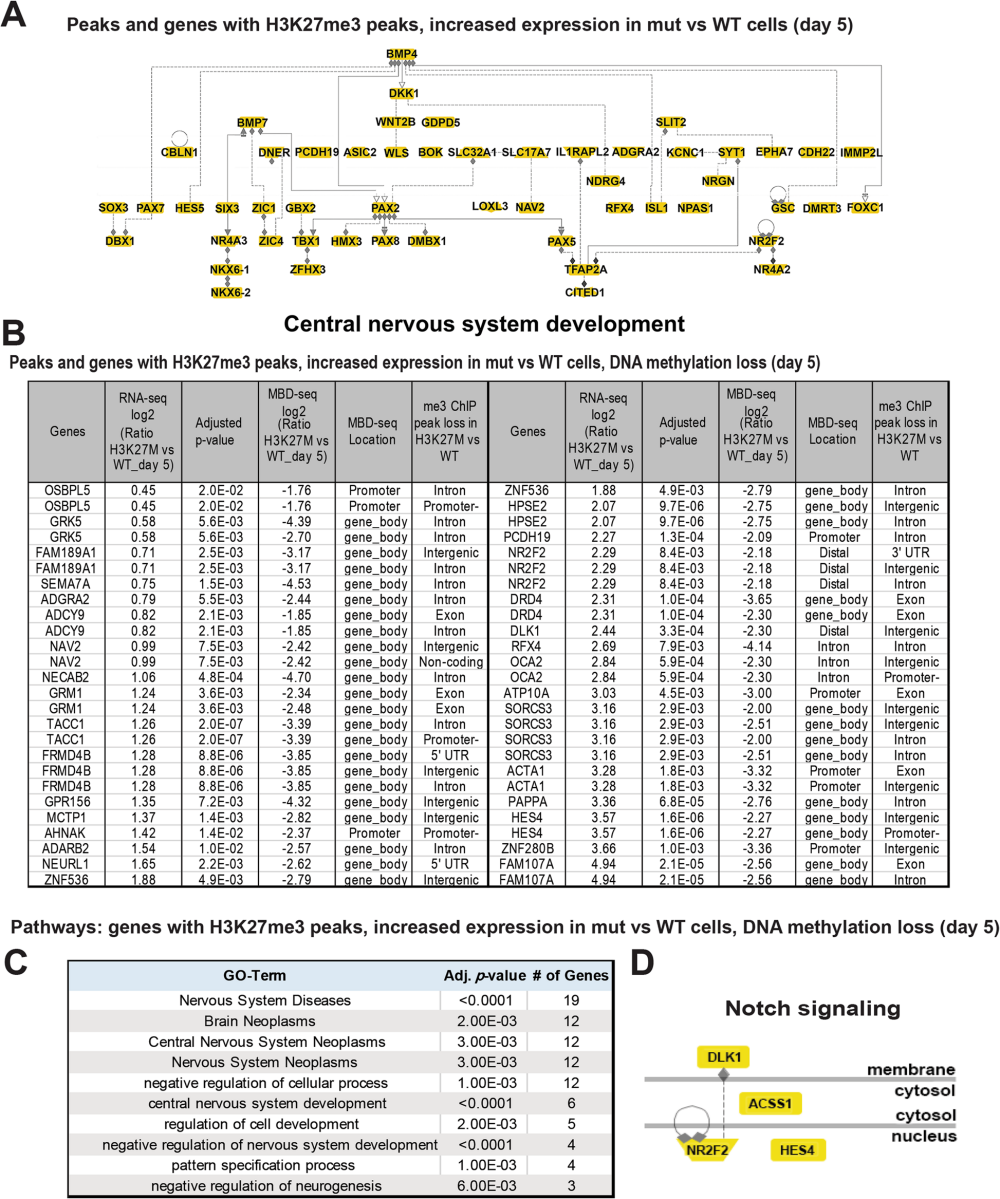
Enriched pathways and gene networks with increased expression in H3K27M cells relative to WT, H3K27me3 peaks, and DNA methylation loss at day 5. **A** Analysis of H3K27me3 peaks at genes with increased expression in mut vs WT at day 5 reveals an enriched network of genes involved in central nervous system development. **B** List of genes with H3K27me3 peaks, increased expression in H3K27M cells relative to WT and DNA methylation loss at day 5. **C** Gene ontology analysis of the genes in **B** reveals enrichment in pathways involved in brain neoplasms, synapses, and Notch signaling. **D** A graphical representation of an enriched network of genes related to Notch signaling

We further integrated these data to examine enriched GO terms in the subset of genes that exhibited more than one of these trends, including genes that exhibited increased expression in mutant versus WT cells and were associated with H3K27me3 peaks (Additional file 10: Table S6), and those with these features and loss of DNA methylation in the day 5 H3K27M cells (Additional file 11: Table S7) [19]. The former set of genes was also associated with a network of genes involved in central nervous system development (Fig. 7A, Additional file 10: Table S6). The latter set represented a relatively small number of peaks and genes (Fig. 7B) but was also associated with related GO terms (Fig. 7C, D, Additional file 11: Table S7). These included brain neoplasms/nervous system neoplasms and other neural-related terms such as central nervous system development, negative regulation of central nervous system development, negative regulation of neurogenesis, and Notch signaling (Fig. 7C, D).

## Discussion

DIPG and more broadly, DMG, are almost exclusively fatal pediatric brain tumors. As a group, they clearly highlight a misalignment between our treatment success in malignant gliomas and our extensive recognition of causative genetic changes and key tumor-promoting events and processes. That H3K27M mutation initiates a path towards DMG is indisputable, but how it does so, beyond altering epigenetic regulation, is unclear. The predilection for these tumors to arise in deep midline brain structures in young children led us to hypothesize that contributory mechanisms to transformation would likely involve targets of epigenetic regulation during early brain development, and might include aspects of neural progenitor clonogenicity, lineage commitment, and differentiation.

We examined each process in human embryonic stem cells and found that, by comparison to control hESCs expressing wildtype H3, those expressing H3K27M mutation exhibited persistent clonogenic activity, which was correlated with greater cell number, colony number, and colony size over time. In addition, the mutation blocked normal differentiation, resulting in abnormal and heterogeneous responses to conditions that, in wildtype cells promote trilineage or neural specification and differentiation. Furthermore, as expected, we found that cells carrying H3K27M exhibited a massive and global loss of H3K27 tri-methylation, which was associated with increased expression of genes involved in cancer, stem cell maintenance, specification, and differentiation toward multiple germ layer-related cell fates. Consistent with the diminished fidelity of differentiation in mutant-expressing clones, there was also a reduction in DNA methylation in H3K27M-expressing cells compared to that in wildtype clones. Together, these data suggest that during early development, H3K27M mutation derails normal differentiation and perpetuates a partially differentiated state with maintenance of clonogenicity. The recent report by Haag et al. also found that H3.3K27M disrupted directed differentiation of iNSCs by preventing normal resolution of bivalent histone marks at critical developmental genes. This may well be the mechanism underlying our observation that H3.3K27M is associated with heterogeneous responses to differentiation cues [20].

Consequently, we investigated whether these transcriptomic differences in our H3K27M versus WT cells are also present in human DIPG tumors versus normal brain. To do so, we mined publicly available microarray data from 35 and 10 DIPG and normal brain tissue samples, respectively [13]. This analysis revealed a concordance in 139 differentially regulated genes and identified a number of commonly dysregulated pathways in our model and in DIPGs involving H3K27M mutation in vivo. These included cAMP signaling pathway, synapse organization and assembly, and epithelial mesenchymal transition (Additional file 12: Table S8).

## Conclusions

There are several mechanisms by which altered differentiation early in neural development together with preserved clonogenicity could increase the likelihood of tumorigenesis. It is widely recognized that cancer risk is relatable to stem cell activity and proliferation [21]. Thus, the persistence of proliferative clonogenic states increases the likelihood of acquiring additional mutations in nascent neomorphic cells. In addition, aberrant differentiation could alter tissue organization and establish a tumor-promoting microenvironment [22, 23]. Both of these are potential consequences of H3K27M mutation and could contribute to the genesis of DMG. Early in development, tissue is replete with growth, migratory, and differentiating factors. The demonstrated loss of a normal response to these developmental cues could result in tissue properties that enhance the acquisition of additional mutations that can cooperate with H3K27M mutation in the genesis of DMG/DIPG. It will be important to analyze this possibility in future research.

Here, we also observed that while H3K27M clones cultured in neural induction media exhibited elevated expression of early neural genes, they failed to acquire the normal neural rosette structure typical of WT cells. These data suggest that tissue disorganization, abnormal elevation of genes associated with early aspects of multilineage fate acquisition, and a subsequent failure to differentiate may contribute to the effects seen here. In vivo, these alterations caused by the H3K27M mutation could favor tumorigenesis if aberrantly differentiated cells disrupt normal deep midline/pontine tissue structure. It was established years ago that abnormal tissue structure is tumor-promoting. Early studies by Bissell and Werb demonstrated that engineered changes in breast duct extracellular matrix alone could promote the development of breast cancer [23]. Similarly, Maffini et al. found that mammary fat pads that had been denuded of epithelial cells and treated with NMU promoted the development of breast carcinomas when wildtype epithelial cells were re-introduced [22]. These studies, along with others, indicate that tissue disorganization can be cancer-promoting and raise the possibility that aberrant differentiation in H3K27M-expressing could result in aberrant tissue structure, which could contribute to tumorigenesis in vivo.

Delineating the mechanisms by which H3K27M mutation predisposes to DIPG/DMG could lead to new treatments for this disease, which currently has a dismal prognosis. Targeting the abnormal histone modification with Panobinostat is currently being evaluated. We did not observe significant differences in DNA methylation corresponding to the loss of H3K27me3, suggesting that DNA methylation gain at these genes may be an event of later development and reliant on H3K27me3 regulation. These findings raise the question of whether targeting the DNA methylation defect with a TET demethylase inhibitor might have a differentiating effect of clinical value.

## Methods

### hESC maintenance and differentiation

H1 human embryonic stem cell (hESC) lines were grown under feeder-free conditions on Matrigel (Corning) in Essential 8™ Flex Medium (A2858501, ThermoFisher Scientific) and regularly passaged as aggregates with ReLeSR (STEMCELL Technologies, Cat. # 05872). hESC colonies exhibiting altered morphology indicating initiation of differentiation were manually removed from the culture; increased propensity to differentiate when grown in stem cell media was a particular characteristic of ESC lines carrying the H3K27M mutation. Experiments adhered to protocols approved by the Washington University hESC Research Oversight Committee (protocol #12-002). As this work was designated nonhuman subjects research by Washington University’s Institutional Review Board, informed consent was not required. Cells were regularly checked for Mycoplasma contamination by using the Mycoalert Mycoplasma Detection Kit, Lonza (Cat. # LT07-118).

### CRISPR/Cas9 genome editing

The H3F3a-K27M-Puro cell line was generated from the H1 human embryonic stem cell (H1 hESC) line (obtained from WiCell) by the Genome Engineering and iPSC Center (GEiC), Washington University in St. Louis. Prior to transfection, the cells were treated with 10 μM Rock (Y-27632) inhibitor for at least 1 h. Approximately 1.5 × 10^6^ H1ESC cells were resuspended in P3 primary buffer and electroporated using a 4D-Nucleofector (Lonza) with 1 μg of H3F3a-K27M-KI-Puromyocin donor plasmid (GeneArt) and 1 μg gRNA SM928.H3F3a.g37 target oligo (5′- atgctggtaggtaagtaagg agg-3′) and 1.2 μg Cas9 expression vector. Nucleofected cells were plated in media containing Rock inhibitor (Y-27632) for 24 h. post nucleofection and then returned to regular E8-Flex media. Following nucleofection, cells were screened with PCR, using primer sets specific to the 5′ and 3′ junctions of the donor oligo and the target gene. After confirmation of donor oligo knock-in in the nucleofected pool, the cells were treated with 0.2 μg/ml of Puromycin for 72 h. Surviving cells were then passaged and treated with 0.5 μg/ml of Puromycin for 72 h and Sanger sequencing was used to confirm the heterozygous knock-in of the K27M mutation in the 5' junction PCR product of the subclones. Karyotyping of the H3F3A-K27M lines was performed by Cell Line Genetics. Five clonal hESC lines with the K27M were utilized for experiments, with all findings documented by 3-6 biological replicate experiments conducted using at least two clonal lines, except for the chromatin immunoprecipitation sequencing (ChIP-seq) analysis, which involved 4 biological replicate experiments performed using one clonal line; the clonal line used for each biological replicate experiment is described in Additional file 2: Table S1.

### Acid extraction of histones and Western blot

Histones were extracted from hESCs and their differentiated derivatives by the acid extraction method, using Abcam’s histone extraction protocol for western blot. Cells were washed with room-temperature phosphate-buffered saline (PBS) and lysed in Triton extraction buffer (TEB; PBS containing 0.5% Triton X-100, 2mM PMSF, 1mM 1, 4-Dithiothreitol (DTT) and protease inhibitors (Roche, Cat. # 11836153001) for 10 min at 4°C. Nuclei were pelleted at 2000 rpm for 10 min at 4°C and washed in TEB before extracting the histones with 0.2 N HCl overnight at 4°C and collecting the supernatant after centrifugation at 2000 rpm for 10 min. Histones were quantified by the colorimetric DC protein assay (Biorad, Cat. # 5000111), according to the manufacturer’s instructions. For Western blotting, 5 μg of protein mixed with NuPAGE LDS sample buffer (ThermoFisher Scientific, Cat. # NP0007) and supplemented with 100 mM DTT was separated on a NuPAGE 4-12 % Bis-Tris protein gel (ThermoFisher Scientific, Cat. # NPO 321) in 1X MES SDS running buffer (ThermoFisher Scientific, Cat. # B0002). Proteins were transferred to nitrocellulose membranes on a trans-blot turbo system and then blocked in Odyssey blocking buffer (Li-COR, Cat. # 927-40000) for 1 h. at room temperature. The blots were then incubated with primary antibody overnight at 4°C with gentle agitation and following four washes in 0.1 % TBST buffer at room temperature, secondary antibodies Donkey anti-Rabbit IRDye 800CW (Green, LI-COR, Cat. # 926-32213) and Donkey anti-Mouse IRDye 600CW (Red, Li-COR, Cat. # 926-68072) diluted in Odyssey blocking buffer were added at room temperature for 1 h. with gentle agitation. Blots were then washed thoroughly with 0.1 % TBST four times and analyzed using the Odyssey Infrared Imaging System (Li-COR). Primary antibodies used for Western blotting were rabbit α-H3K27M (1:1000, Millipore, Cat. # ABE419) and mouse α-H3 (pan histone) (1:2000, Cell Signaling Tech, Cat. # 36381).

### Clonogenic or extreme limiting dilution assays (ELDA analysis)

The frequency of clonogenic sphere formation ability of WT and H3K27M H1 hESCs was assayed by the extreme limiting dilution assay (ELDA) as described in Kfoury et al., *Acta Neuropathol*., 2018 [24]. Briefly, hESC cells were dissociated with Accutase into a single cell suspension and plated in 96-well ultra-low attachment plates with E8 Flex media in a serial dilution, ranging from 3000 cells/well to 1 cell/well (3000, 600, 120, 24, 5, and 1 cells; *n* = 14/cell density). Sphere formation was measured 7 days after plating. Clonogenic stem-like cell frequency was analyzed using the Extreme Limiting Dilution Analysis software (http://bioinf.wehi.edu.au/software/elda/).

### ATP viability assay for 3-D hESC spheres

About 10,000 H1 hESCs were plated per well in ultra-low attachment 96-well plates in E8 Flex media for 72 h at 37 °C. After 3D spheres were formed, 100 μL of CellTiter-Glo Reagent (Promega, Cat. # G9681) was added, and samples were triturated 10X, followed by 15 min incubation at room temperature (RT). Plates were shaken for an additional 2 min in the plate reader and luminescence was measured in a Tecan i-control plate reader. Data is normalized to a mock control and six independent biological replicates were performed with 3 technical replicates for each of the biological replicate.

### hESC differentiation into cortical neural progenitor cells (NPCs)

hESCs were dissociated with Accutase to single cells, counted, and 3.5 million cells per well were seeded into each well (with 300 microwells per sample type generated) of anti-adherent AggreWell™800 Plates (Stem Cell Technologies, Cat. # 34815; cell number was within the manufacturer’s recommended range for each well of the AggreWell 800 plate) to produce uniform size embryoid bodies (EBs) containing approximately 11,666 cells per EB. AggreWell plates were pre-rinsed with warm neural induction medium (NIM) before plating the single cell suspension of H1 hESCs (WT and H3K27M mutant) and the plates were spun at 100 × *g* for 3 min to capture the cells into the microwells and were then incubated at 37°C with 5% CO2 in NIM medium for 2 days for the EB formation. NIM medium composition was based on published protocols [25–27] and consisted of Neurobasal-A (Life Technologies), 1X B27 supplement without Vitamin A (LifeTechnologies, Cat. # 12587010), 10 μM SB-431542 (Tocris Biosciences), 100 nM LDN-193189 (Stemgent), and 10 μM Y-27632 (Tocris Biosciences). On day 2, EBs were removed from the AggreWell 800 plates and seeded onto poly-L-ornithine (20μg/mL)- and laminin (10μg/mL)-coated plates, enabling them to generate neural rosettes. Medium was replenished every other day and NPCs of telencephalic regional character were isolated on day 5 using Accutase (Gibco, Cat. # A1110501).

### Mesodermal, endodermal, and ectodermal differentiation in defined media

The functional ability of hESCs to differentiate into three germ layers was tested with defined media conditions by using the STEMdiff™ Trilineage Differentiation Kit (STEM CELL Technologies, Cat. # 05230) according to the manufacturer’s instructions with slight modifications to cell numbers plated. Briefly, hESCs were disaggregated with Accutase, counted, and plated at densities of 2×10^5^, 1.5×10^5^, and 2×10^5^ cells per well for ectoderm, mesoderm, and endoderm differentiation respectively, in separate Matrigel-coated wells of a 12-well plate. The ectoderm differentiation medium with 10 μM Rock inhibitor (Y-27632, Cat. # 72302, STEM Cell inhibitor) was added on day 0 and the cells were maintained in ectoderm differentiation medium without Rock inhibitor from day 1 until the cell harvest on day 4 with Accutase. The cells in the rest of the wells were plated with E8 Flex medium with 10 μM Rock inhibitor and Rock inhibitor was removed on day 1. Mesoderm and endoderm differentiation medium (without Rock inhibitor) was added to respective wells on day 2 and harvested on day 4.

### Protein arrays

The Human Pluripotent Stem Cell Antibody Array (R&D Systems, Cat. # ARY010) was used to analyze relative expression of 15 protein markers (including markers of stem cells and the three primary germ layer markers) in the pluripotent H1 hESCs and their differentiated lineages obtained from trilineage differentiation. A commercial protein assay kit (Biorad, Cat. # 5000111) was used to analyze the levels of protein markers on the Human Pluripotent Stem Cell Antibody Arrays and was performed according to the manufacturer's instructions. In brief, cells were collected and lysed in the lysis buffer provided with the kit and supplemented with proteinase inhibitors (Roche, Cat. # 11836153001) and 100 μg of total cell protein was used to probe protein arrays to quantitate the levels of each marker protein in the lysates. Protein arrays were developed using SuperSignal West Femto Maximum Sensitivity Substrate (Thermo Fisher Scientific) and were imaged using the BioRad chemiluminescence imaging system. Signal intensity of each spot was quantified with Quick Spots (Western Vision Software) image analysis software and normalized to internal standards on each array [28].

### Trilineage differentiation potential

The potential of the WT and H3K27M hESC lines to undergo differentiation into the primary germ layers and their derivatives was also tested by cell specification in KnockOut Serum Replacement (KOSR) medium containing 80% DMEM (Gibco, Cat. # 21331020), 20% KnockOut Serum Replacement (Gibco, Cat. # 10828-028), 1x non-essential amino acids (Gibco, Cat. # 11140-050), 1x GlutaMAX™-I (Gibco, Cat. # 35050-079) and β-mercaptoethanol (0.1 mM). hESCs were dissociated into single cells with Accutase (Gibco, A1110501) and approximately 0.5 million cells per well were seeded into each well of an anti-adherent AggreWell™800 Plate (Stem Cell Technologies, Cat. # 34815) pre-rinsed with warm KOSR medium. Single-cell suspensions of H1 hESCs (WT and H3K27M mutant) were plated and spun at 100*g* for 3 min to collect cells into the microwells and were incubated at 37 °C with 5% CO2 in KOSR medium for 2 days for EB formation. On day 2, EBs were removed from the AggreWell 800 plates and seeded onto Geltrex-coated plates (ThermoFisher Scientific, Cat. # A1413201; 1:100 dilution in pre-chilled (4 °C) DMEM/F-12 medium; 1× final concentration), for testing trilineage differentiation.

### hESC scorecard assay

For the hESC Scorecard Assay, RNA from hESCs and cells differentiated in KOSR was isolated using the QIAshredder (Qiagen, Cat. # 79654) and the RNeasy Plus Mini kit (Qiagen, Cat. # 74134) following the manufacturer’s instructions. For cDNA preparation, 1 μg of total RNA/sample was used for cDNA synthesis with the High-Capacity cDNA Reverse Transcription Kit (ThermoFisher Scientific, Cat. # 4368814). hESCs Scorecard assays (Life Technologies Cat. # A15871) were run according to the manufacturer’s instructions on the template that is compatible with StepOne Plus RT-PCR System (Applied Biosystems), with normalization and fold differences between WT and H3K27M models determined by using the accompanying hPSC Scorecard Analysis Software.

### Mouse immunohistochemistry

RCAS mice, P53flox/flox homozygous, Nestin-TVa+ GEMMs of DIPG model were used in accordance with National Institutes of Health guidelines following protocols approved by the Washington University Animal Studies Committee (protocol number 20180206). To generate the model, DF-1 chick fibroblast cells (ATCC CRL-12203) were transfected with one of three RCAS expression plasmids encoding H3.3K27M, PDGF-B, and Cre, with FuGENE 6 transfection reagent (#E2311, Promega, Madison, WI). After at least 3 passages and once confluent, cells were harvested in a 1:1:1 H3.3K27M:PDGFB:Cre ratio in medium and injected into the brainstem of postnatal day 7 mice (0.6~0.8 million cells/in 1.2 μl/mouse). Tumors formed within 4 weeks. Age-matched RCAS mice without injection were used as controls. Mice were perfused with PBS and 4% PFA, and brain tissue was harvested for paraffin sectioning (8μm sections). Sections were de-paraffinized, and antigen retrieval was performed at 95°C in 10mM sodium citrate (pH=6.0) for 20 mins, followed by blocking for 1 h at room temperature and immunostaining for the following antibodies: rabbit anti-H3K27M (Sigma SAB5600095), rabbit anti-Hes5 (Invitrogen MA5-38214), rat anti-GFAP (Invitrogen 13-0300), mouse anti-Olig2 (Sigma MABN50), mouse NeuN (Millipore MAB377), and rat anti-Ki67 (Invitrogen 14-5698-82).

### RNA-Seq

RNA from H1 hESC and their differentiated derivatives was isolated with RNeasy Plus Mini kit (Qiagen, Cat. # 74134) using QIAshredder (Qiagen, Cat. # 79654) and on-column digestion of genomic DNA following manufacturer’s recommendations. The kit captures RNA molecules higher than 200 nucleotides in length, effectively removing smaller RNAs such as 5.8S rRNA, 5S rRNA, and tRNAs. For RNA-Seq, RNA was initially quantified by NanoDropND-1000 spectrophotometer (Thermo Scientific) and the concentration/quality of total RNA was further confirmed on the Agilent Bioanalyzer2100. Only samples that had an RNA integrity number (RIN) > 8 were used for RNA-seq studies. RNA-seq library preparation and Illumina sequencing were performed by the Genome Technology Access Center (GTAC) at Washington University School of Medicine. Total RNA was enriched for polyA-tailed mRNA and mRNA fragmentation, first- and second-strand cDNA synthesis, and the addition of indexed adapters was carried out as previously described [29].

### RNA-Seq analysis

Single-end, 50 nt reads were obtained on an Illumina HiSeq 3000 instrument, and the sequencing reads were mapped to the human genome (hg19 UCSC) using HISAT2 [30]. The mean number of transcriptome-aligned reads per sample was 17.2 million. Read quantitation on a per-gene basis was performed with HTSeq [31]. Detectable RNAs were assessed as those present at or above 1 read per million (18 reads) in at least 2 of 4 samples from any treatment group, resulting in 16,083 RNAs for downstream analyses. Calculation of fold-change and false discovery rate (FDR) for differentially expressed RNAs was performed using the R/Bioconductor packages limma and edgeR/voom [32]. Partek Genomics Suite v6.6 (Partek, St. Louis, MO) was used for inter-sample quality control by principal components analysis and for heat map construction. To visualize differentially expressed mRNAs, normalized read counts from edgeR were subjected to unsupervised hierarchical clustering with Euclidean distance and average linkage using Partek Genomics Suite v6.6. Unless otherwise specified, standardized heatmaps are shown in which map colors represent the extent of deviation from the raw average. For clarity of presentation and to enable comparison with other studies, individual gene expression levels in column graphs are rendered as FPKM (Fragments Per Kilobase of exon per Million reads mapped to the transcriptome), calculated from Tophat-aligned read counts using Cufflinks 2.1.1 [33]. For visualization of reads on the WashU epigenome browser, RNA-seq reads were aligned to the human genome (assembly hg38) with STAR version 2.4.2a [34].

### RT-qPCR validation of RNA-seq findings

For RT-qPCR, 1μg total RNA was reverse transcribed using iScript Reverse Transcription Supermix (Bio-Rad) and equal quantities of cDNA were used as a template for RT-qPCR using the Applied Biosystems Fast Real-Time quantitative PCR platform. RPL30 was used as an endogenous control for normalization.

### Methyl-CpG binding domain sequencing (MBD-seq)

Methyl-CpG binding domain sequencing (MBD-seq) was performed on six biological replicates of WT and H3K27M mutant undifferentiated H1 hESCs and their 5-day differentiated neural EB derivatives cultured in parallel to those used for RNA-sequencing. Genomic DNA was isolated from the harvested cells using the PureLink™ Genomic DNA Mini Kit (ThermoFisher Scientific, K182002) and ~3 μg of DNA was fragmented to 100- to 500-bp fragments in a water bath sonicator (Bioruptor Pico, Diagenode) using 30 sec on/off cycles for 10 min. MBD2 enrichment was performed with the MethylMiner™ Methylated DNA Enrichment Kit (ThermoFisher Scientific, Cat. # ME10025). Approximately 1-2 μg of fragmented genomic DNA was incubated with human MBD2-biotin protein coupled to paramagnetic Dynabeads® M-280 Streptavidin for methylated CpG enrichment according to the manufacturer’s protocol and the methylated CpG fragments were finally eluted twice with high salt (2000 mM NaCl) elution buffer. The enriched DNA was purified and concentrated by ethanol precipitation and the DNA concentration was measured with Quant-iT™ dsDNA HS (High Sensitivity) Assay (ThermoFisher Scientific, Cat. # Q32854). Enrichment of methylated DNA with the MBD2 pulldown was confirmed by qPCR of the methylated positive control region of BRD1 and the unmethylated negative control region of CoQ3 ([35] and oligo sequences provided in Additional file 2: Table S1). Enriched DNA was then sent to the GTAC sequencing core for library preparation and sequencing. Briefly, MBD captured DNA fragments were run on the Agilent bioanalyzer high sensitivity chips to assay size range. 10ng of DNA as determined by Qubit was blunt ended, had addition of “A” base to 3′ end, and had sequencing adapters ligated to the ends. The fragments were size selected to 200–600 base pairs and underwent amplification for 15 cycles with primers incorporating p5 and p7 sequences and a unique index tag for multiplexing. The resulting libraries were sequenced using the Illumina HiSeq3000 as paired reads extending 150 bases. Positive and negative control amplicons used to validate that methylated DNA precipitation was successful are shown in Additional file 2: Table S1.

### MBD-seq analysis

Sequenced reads were mapped to the human genome (hg19 UCSC) using HISAT2 [30] and the resulting bam files were analyzed for methyl-binding domain peaks with the software package MEDIPS version 1.30.0 under Bioconductor 3.6 [36]. A window size of 150 bp with a minimum fragment count (number of consecutive CpG sites) of 5 per window, an extend parameter (read gap distance) of 300, and a Benjamini–Hochberg false discovery rate, FDR<0.1 were used for peak determination. Peaks corresponding to differentially methylated regions from pairwise comparisons were defined and annotated for occurrence within gene bodies with MEDIPS. For peaks occurring in intergenic, promoter, or enhancer regions, annotation was performed with the bedtools package (intersect command) (http://bedtools.readthedocs.io/en/latest/index.html) and bed files derived from the UCSC genome browser corresponding to Broad ChromHMM, CpG island, and NCBI RefSeq annotations. For visualization of data on the Epigenome Browser, liftOver (UCSC Browser) was used to move differential methylation peaks to hg38.

### ChIP-seq

H1 hESCs (WT and H3K27M) were collected and fixed with paraformaldehyde (1% final concentration, Electron Microscopy Sciences, Cat. #15710) and quenched with 125 mM Glycine. Cell pellets were resuspended in 3 ml cell lysis buffer (5 mM PIPES pH 8.0, 85 mM KCl, 0.5% IPGAL including protease inhibitors) and incubated on ice for 10 min, and then spun at 5000 rpm for 5 min, and the supernatant discarded. Nuclei were resuspended in 1 ml nuclear lysis buffer (50 mM Tris.HCl pH8.0, 10 mM EDTA, 1% SDS plus protease inhibitors) then sonicated with a Biorupter (Diagenode) 30 s on/off, repeated for 12 cycles in total. The sheared chromatin was confirmed to be between 300 and 500 bp by agarose gel electrophoresis, and the samples were stored at − 80°C until processed. 100 μg of chromatin was diluted in ChIP dilution buffer (16.7 mM Tris–HCl (pH 8.1), 167 mM NaCl, 0.01% SDS, 1.1% Triton X-100, 1.2 mM EDTA), and incubated overnight at 4°C with primary antibodies H3K27me3 (Millipore Sigma, Cat. #07-449), and IgG (Millipore Sigma, Cat. #PP64-10-KC) in the cold room. The next day, Protein A Dynabeads (Invitrogen) were washed three times in ChIP dilution buffer and added to the chromatin, and then the incubation was further continued for 4 h. After incubation, the beads were washed sequentially with 1× low-salt buffer (0.1% SDS, 1% Triton X-100, 2 mM EDTA, 20 mM Tris (pH 8), 150 mM NaCl], 1× high-salt buffer (0.1% SDS, 1% Triton X-100, 2 mM EDTA, 20 mM Tris (pH 8), 500 mM NaCl), 2× LiCl buffer (0.25 M LiCl, 1% IGEPAL CA- 630 (Sigma), 1% sodium deoxycholate, 1 mM EDTA, 10 mM Tris, pH 8.0). Bead pellets were resuspended in 125 μl of elution buffer (1% SDS, 5 mM DTT, and 150 mM NaCl in Tris-EDTA) and incubated at 65 °C for 10 min. The supernatant was collected in a new tube, and the elution was repeated again. DNA was reverse cross-linked (with 200mM NaCl, 1% SDS, 5 mM DTT) at 65°C overnight followed by Proteinase K digestion (250 μg Proteinase K) at 37°C for 1 h. The DNA samples were purified using the NucleoSpin gel/PCR purification kit (Takara). DNA sizing was determined using Agilent Bioanalyzer, and concentration was determined with Qubit assay. Library preparation was performed with up to 10 ng of DNA. DNA was blunt ended, had an “A” base added to the 3′ ends, and then had Illumina sequencing adapters ligated to the ends. Size selection was performed with Ampure XP beads (Beckman Coulter) to select for fragments between 150 and 700bp. Ligated fragments were then amplified for 15 cycles using primers incorporating unique dual index tags. Fragments were sequenced on an Illumina NovaSeq-6000 using paired-end reads extending 150 bases.

### ChIP-seq analysis

ChIP-seq data analysis was performed as described previously [37]. Briefly, raw reads were aligned to the human genome (assembly hg38) with the Burrows–Wheeler Aligner (BWA) [38]. The alignment results (bam) were processed to bed and BigWig format by using methylQA [39]. MACS2 [40] was used to call the peaks by using H3K27me3 ChIP against IgG control with a q-value of less than 0.01 in narrow peak mode. The peaks located in blacklist regions were removed. The recurrent peaks (identified in at least three of four biological replicates with a minimum 50% overlap of each other) were further merged and used for downstream analysis.

## Supplementary Information

**Additional file 1: Figure S1.** Derivation of hESC lines carrying the H3K27M mutation. (A) CRISPR-Cas9-mediated genome engineering was used for knock-in of a single base mutation (A>T) into one allele of the H3F3A gene in the H1 hESC line, resulting in cells heterozygous for mutant H3F3A, which encodes an H3.3 protein with a K to M amino acid substitution at position 27 (H3K27M). Multiple independent clonal lines carrying this mutation were derived, with sequence analysis of three clonal lines (C1-3) shown. (B) Sanger sequencing was used to confirm that this mutation was present in each clone. (C) Clonal hESC lines with H3K27M mutation were confirmed to have a normal karyotype. (D) Clonal hESC lines were all demonstrated to express H3K27M protein, by western blotting isolated histones with antibodies specific to the mutated variant of H3.3 (H3K27M) or to pan-H3 (as a loading control). (E) Original and uncropped images of the western blot gels presented in panel (D).

**Additional file 2: Table S1.** Biological replicates and clonal lines used. (A) Biological replicate experiments performed, and clones used for each biological replicate experiment are shown. Results obtained for the wild type (WT) H1 hESC line were compared with at least two different clonal H1 derivatives with CRISPR-mediated introduction of the H3K27M mutation, across 3-6 biological replicate experiments, except for ChIP-seq, which involved H3K27M clone 1 versus WT comparisons. (B) ELDA assay: biological replicate experiments performed, and clones used for each biological replicate experiment as well as individual data values are shown. (C) Titer-Glo assay: biological replicate experiments performed, and clones used for each biological replicate experiment as well as individual data values are shown. (D-L) Protein assay: biological replicate experiments performed, and clones used for each biological replicate experiment as well as individual data values are shown. (M-O) Scorecard assay: biological replicate experiments performed, and clones used for each biological replicate experiment as well as individual data values are shown. (P) RT-qPCR assay: biological replicate experiments performed, and clones used for each biological replicate experiment as well as individual data values are shown. (Q) Positive and negative control primer pairs used for DNA methylation analysis.

**Additional file 3: Figure S2.** NeuN and H3K27M immunostaining. Representative immunostaining image for the indicated proteins in RCAS DMG tumor tissue sections are shown. Scale bar=50μm.

**Additional file 4: Table S2.** Transcriptomic analysis of differential gene expression in the K27M (mutant) versus wild type cells. Differentially expressed genes in clusters a-e are shown, with their fold changes and *p-*value/adjusted *p-*value. Final sheet shows differential gene expression (log2 fold change) and adjusted *p-*values for each pairwise sample comparison.

**Additional file 5: Figure S3.** RT-qPCR validation of findings from RNA-seq. Genes from Figure 4D, cluster b, which exhibited the strongest trend in the RNA-seq analysis, being upregulated in H3K27M mutant versus WT cells at day 5, were selected for validation. This cluster of genes was highly enriched for genes involved in glioblastoma, tumor angiogenesis or progression, and neural stem cells. RT-qPCR was used to compare mRNA expression levels in the mutant versus wild type cells. Four biological replicate experiments were conducted in technical triplicate using one clonal line. *** *p*<0.0001 was determined by two tailed student's t-test. Clones used for each biological replicate experiment and numbers of biological replicate experiments performed are described in Additional file 2: Table S1.

**Additional file 6: Table S3.** Pathway analysis of differentially expressed genes defined enriched gene ontology (GO) terms. DEGs from clusters a-e were analyzed. (PPTX 41 kb)

**Additional file 7: Figure S4.** Networks of genes with elevated expression in the H3K27M cells, relative to WT, at day 5. Analysis of differentially expressed genes in Figure 4 cluster b, which showed elevated expression in the mutant cells at day 5, revealed enriched networks of genes related to (A) angiogenesis, (B) cell invasion, and (C) cell proliferation.

**Additional file 8: Table S4.** ChIP-seq analysis demonstrates widespread loss of H3K27me3 in H3K27M mutant cells. (A) Numbers of peaks meeting each criterion, (B) all peaks obtained in either H3K27M mutant or WT cells, and those peaks that were unique (non-intersecting) between the two sample types. (C) Genes associated with H3K27me3 peaks that were unique to the WT sample. (D-F) GO terms associated with genes that were unique to the WT sample. (G) Genes associated with H3K27me3 peaks that were unique to the H3K27M mutant sample. (H-J) GO terms associated with genes that were unique to the mutant sample.

**Additional file 9: Table S5.** Analysis of differential DNA methylation in H3K27M mutant versus WT cells. (A) DNA methylation peaks gained in the H3K27M mutant versus WT samples at day 0. (B-D) GO terms associated with day 0 H3K27M mutant gained peak-associated genes. (E) DNA methylation peaks lost in the mutant vs WT samples at day 0. (F-H) GO terms associated with day 0 mutant lost peak-associated genes. (I) DNA methylation peaks gained in the mutant vs WT samples at day 5. (J-L) GO terms associated with day 5 mutant gained peak-associated genes. (M) DNA methylation peaks lost in the mutant vs WT samples at day 5. (N-P) GO terms associated with day 5 mutant lost peak-associated genes.

**Additional file 10: Table S6.** GO terms associated with genes with increased expression in the mutant versus WT at day 5 and H3K27me3 at day 0. Enriched biological processes, cellular components, molecular functions, and MeSH disease terms are shown with the *p-*value/adj. *p-*value (FDR), genes observed versus expected, and term-associated genes.

**Additional file 11: Table S7.** GO terms associated with genes with increased expression in the mutant versus WT at day 5, H3K27me3 at day 0, and loss of DNA methylation in the mutant at day 5. Enriched biological processes, cellular components, molecular functions, and MeSH disease terms are shown with the *p-*value/adj. *p-*value (FDR), genes observed versus expected, and term-associated genes.

**Additional file 12: Table S8.** Concordance in gene expression patterns between our human embryonic stem cells with H3K27M mutation and DIPG tumors involving H3K27M mutation. (A) Pathway enrichment analysis for concordant differentially regulated genes in H3K27M versus WT cells at day 5 and DIPG tumor versus normal brain tissue samples. (B) List of concordant differentially regulated genes (139 genes) in H3K27M versus WT cells at day 5 and DIPG tumor versus normal brain tissue samples.

## Data Availability

All data generated or analyzed during this study are included in this published article, its supplementary information files, and publicly available repositories. Clones used for each biological replicate experiment and numbers of biological replicate experiments performed are described in Additional file 2: Table S1 for each finding in this manuscript. All raw data and processed files for RNA-seq, MBD-seq, and ChIP-seq have been deposited in the Short Read Archive/Gene Expression Omnibus database (https://www.ncbi.nlm.nih.gov/geo/) as GSE159071 SuperSeries record.

## Abbreviations

H3K27M: Lysine 27 to methionine mutation
DIPGs: Diffuse Intrinsic Pontine Gliomas
H3K27me3: H3K27 trimethylation
DMG: Diffuse Midline Gliomas
PRC2: Polycomb repressive complex 2
H3K27ac: H3K27 acetylation
hESC: Human embryonic stem cell
ELDA: Extreme limiting dilution assay
WT: Wild type
EBs: Embryoid bodies
PLO: Poly-L-ornithine
KOSR: KnockOut Serum Replacement
DEGs: Differentially expressed genes
PCA: Principal component analysis
GO: Gene ontology
MBD-seq: Methyl-CpG binding domain sequencing
GEiC: Genome Engineering and iPSC Center
ChIP-seq: Chromatin immunoprecipitation sequencing
PBS: Phosphate-buffered saline
TEB: Triton extraction buffer
DTT: 1, 4-Dithiothreitol
RT: Room temperature
NPCs: Cortical Neural Progenitor Cells
GTAC: Genome Technology Access Center
hg19 UCSC: Human genome
FDR: False discovery rate
FPKM: Fragments Per Kilobase of exon per Million
BWA: Burrows–Wheeler Aligner
PSC: Pluripotent stem cell
