# Supplementary figures and images for: The H3K27M mutation alters stem cell growth, epigenetic regulation, and differentiation potential
Source: BMC Biol. 2022 May 30;20:124. doi: 10.1186/s12915-022-01324-0 (PMC9153095; doi:10.1186/s12915-022-01324-0)

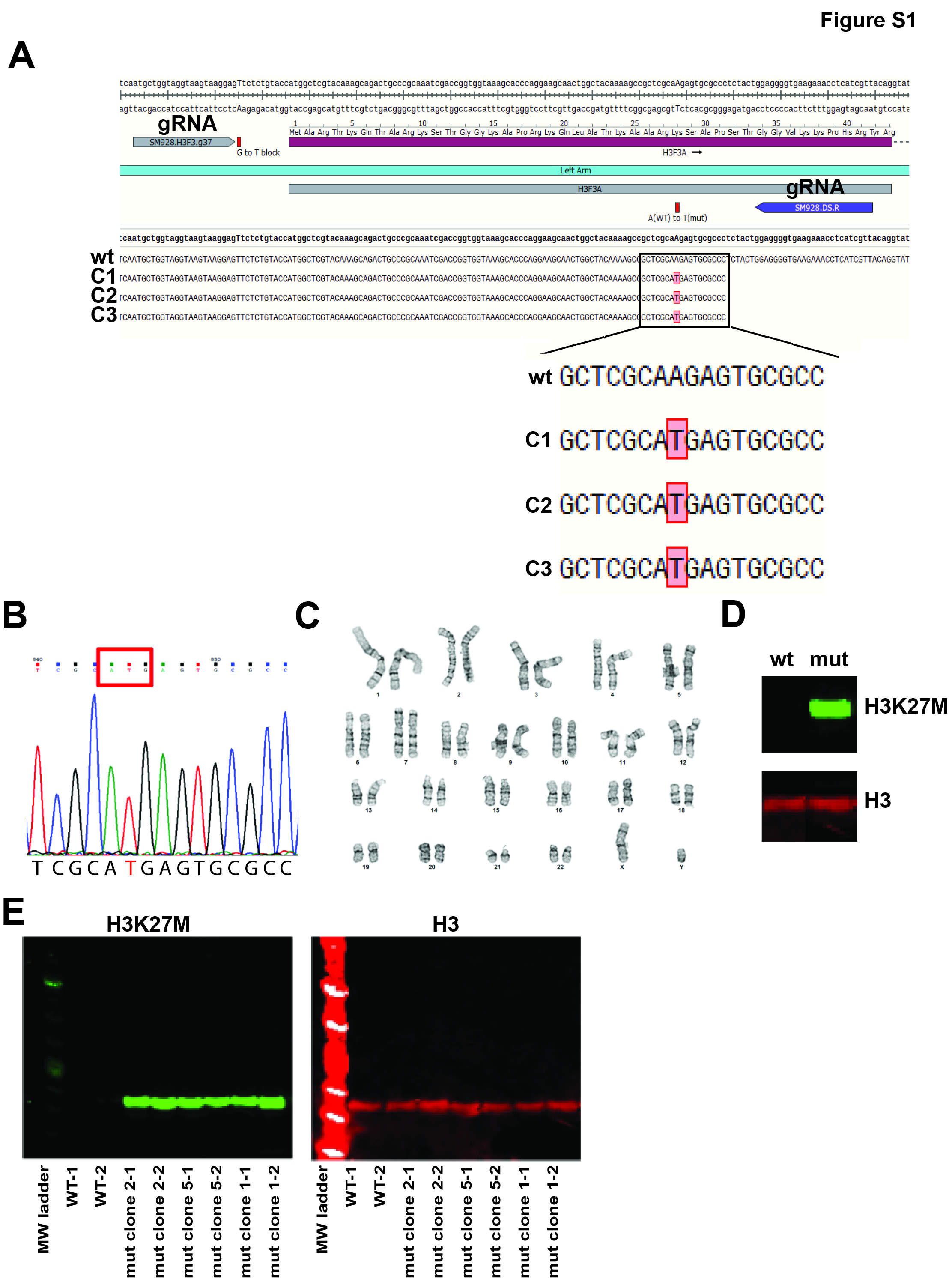

Supplement: Supplementary file 1 — Additional file 1: Figure S1. Derivation of hESC lines carrying the H3K27M mutation. (A) CRISPR-Cas9-mediated genome engineering was used for knock-in of a single base mutation (A>T) into one allele of the H3F3A gene in the H1 hESC line, resulting in cells heterozygous for mutant H3F3A, which encodes an H3.3 protein with a K to M amino acid substitution at position 27 (H3K27M). Multiple independent clonal lines carrying this mutation were derived, with sequence analysis of three clonal lines (C1-3) shown. (B) Sanger sequencing was used to confirm that this mutation was present in each clone. (C) Clonal hESC lines with H3K27M mutation were confirmed to have a normal karyotype. (D) Clonal hESC lines were all demonstrated to express H3K27M protein, by western blotting isolated histones with antibodies specific to the mutated variant of H3.3 (H3K27M) or to pan-H3 (as a loading control). (E) Original and uncropped images of the western blot gels presented in panel (D). [file 12915_2022_1324_MOESM1_ESM.tif]

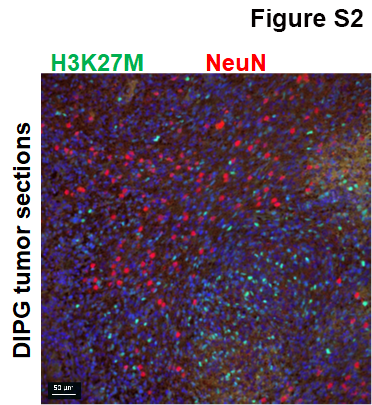

Supplement: Supplementary file 3 — Additional file 3: Figure S2. NeuN and H3K27M immunostaining. Representative immunostaining image for the indicated proteins in RCAS DMG tumor tissue sections are shown. Scale bar=50μm. [file 12915_2022_1324_MOESM3_ESM.tif]

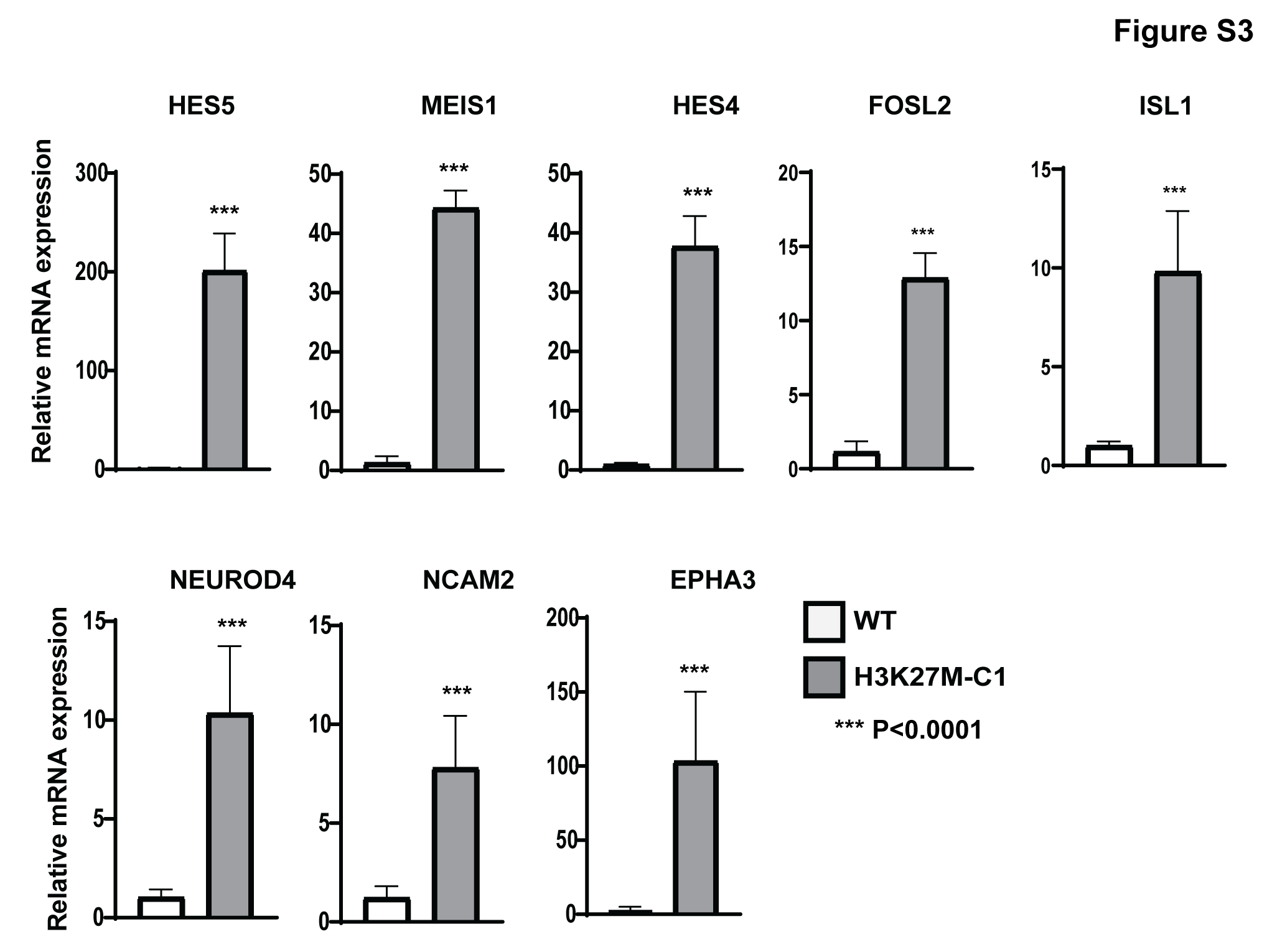

Supplement: Supplementary file 5 — Additional file 5: Figure S3. RT-qPCR validation of findings from RNA-seq. Genes from Figure 4D, cluster b, which exhibited the strongest trend in the RNA-seq analysis, being upregulated in H3K27M mutant versus WT cells at day 5, were selected for validation. This cluster of genes was highly enriched for genes involved in glioblastoma, tumor angiogenesis or progression, and neural stem cells. RT-qPCR was used to compare mRNA expression levels in the mutant versus wild type cells. Four biological replicate experiments were conducted in technical triplicate using one clonal line. *** p<0.0001 was determined by two tailed student's t-test. Clones used for each biological replicate experiment and numbers of biological replicate experiments performed are described in Additional file 2: Table S1. [file 12915_2022_1324_MOESM5_ESM.tif]

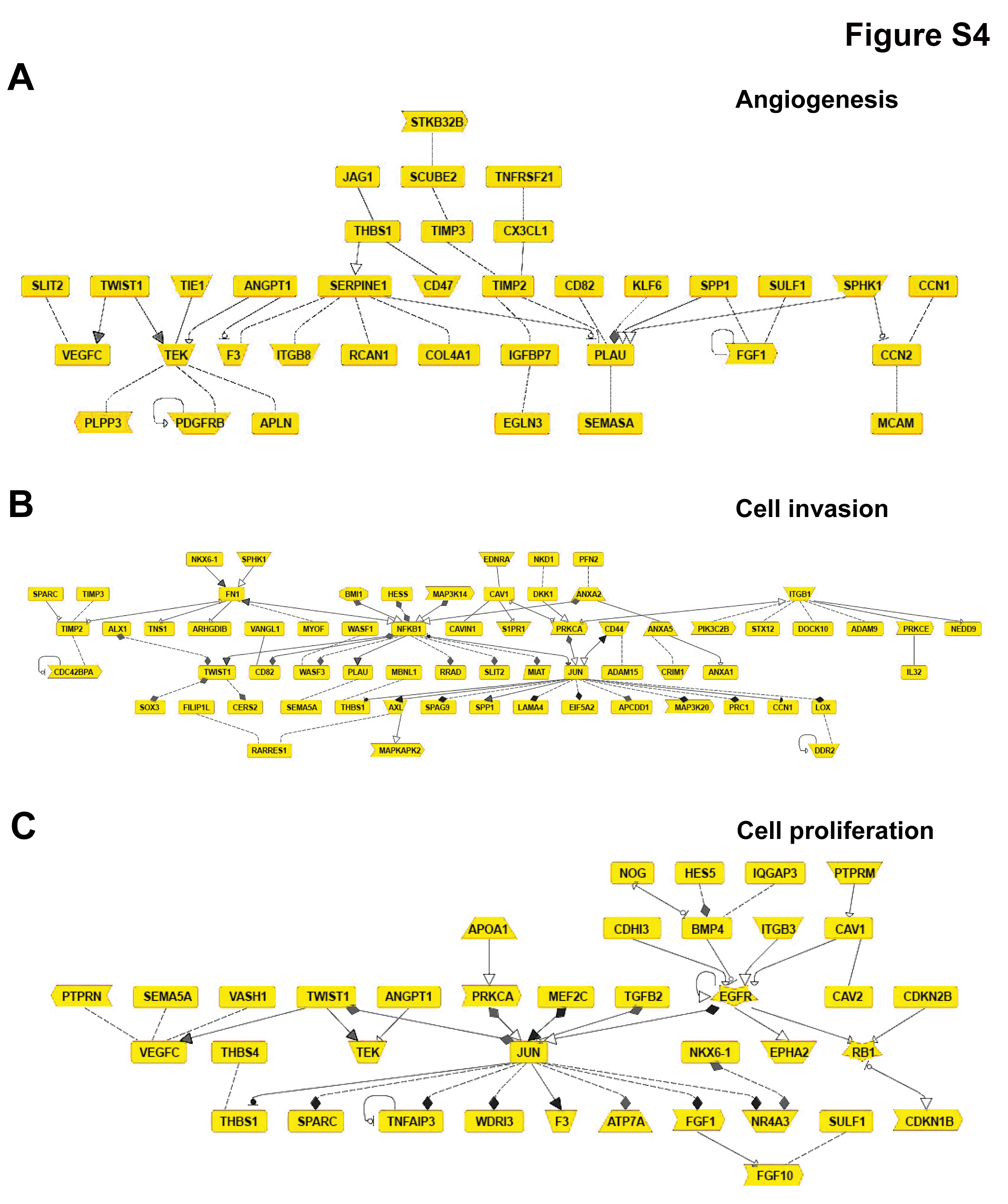

Supplement: Supplementary file 7 — Additional file 7: Figure S4. Networks of genes with elevated expression in the H3K27M cells, relative to WT, at day 5. Analysis of differentially expressed genes in Figure 4 cluster b, which showed elevated expression in the mutant cells at day 5, revealed enriched networks of genes related to (A) angiogenesis, (B) cell invasion, and (C) cell proliferation. [file 12915_2022_1324_MOESM7_ESM.tif]
