# Supplementary material for: The H3K27M mutation alters stem cell growth, epigenetic regulation, and differentiation potential
Source: BMC Biol. 2022 May 30;20:124. doi: 10.1186/s12915-022-01324-0 (PMC9153095; doi:10.1186/s12915-022-01324-0)
Supplement: Supplementary file 6 — Additional file 6: Table S3. Pathway analysis of differentially expressed genes defined enriched gene ontology (GO) terms. DEGs from clusters a-e were analyzed. (PPTX 41 kb) [file 12915_2022_1324_MOESM6_ESM.pptx]

## Slide 1
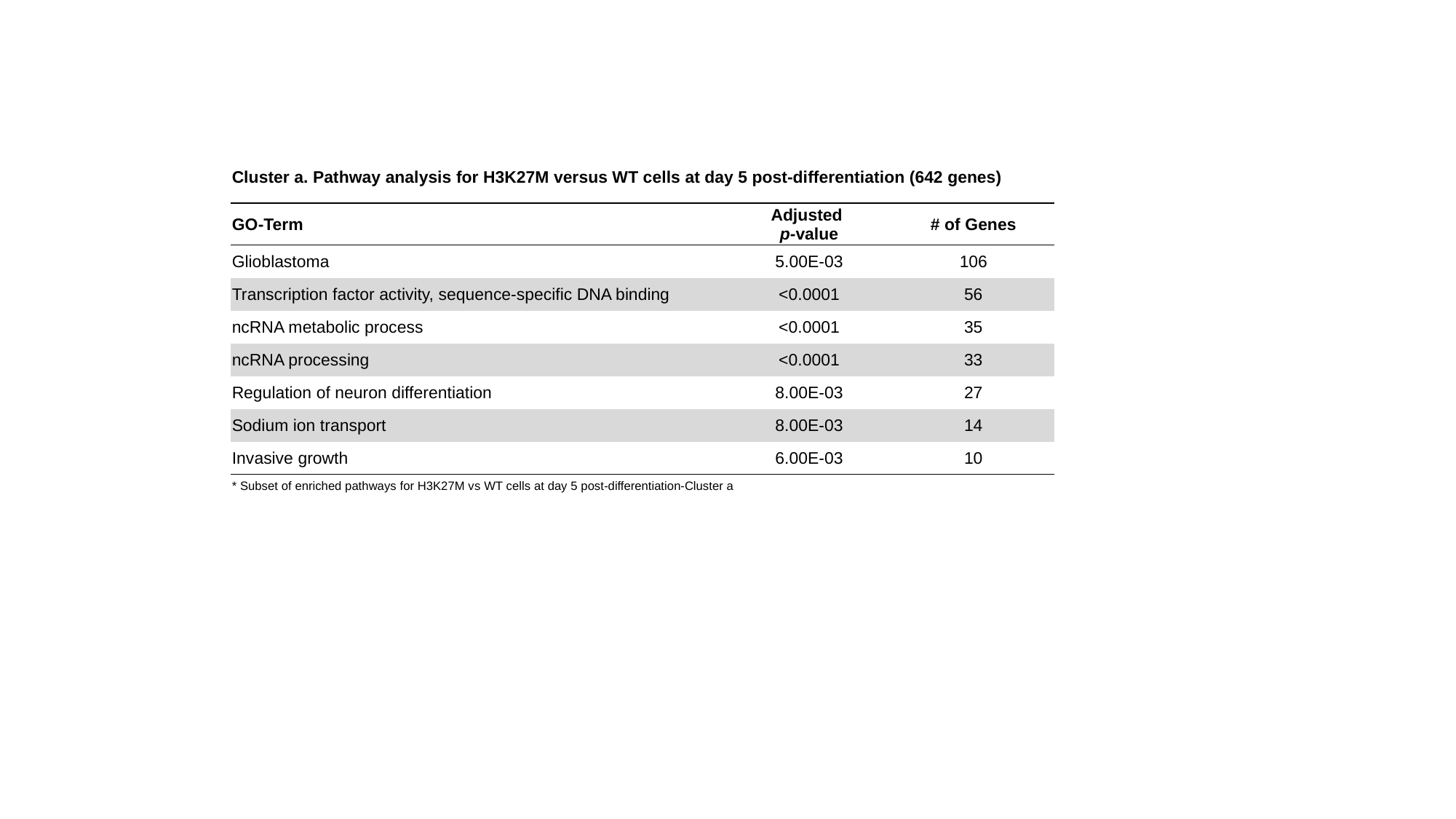

| Cluster a. Pathway analysis for H3K27M versus WT cells at day 5 post-differentiation (642 genes) | | |
| --- | --- | --- |
| GO-Term | Adjusted p-value | # of Genes |
| Glioblastoma | 5.00E-03 | 106 |
| Transcription factor activity, sequence-specific DNA binding | <0.0001 | 56 |
| ncRNA metabolic process | <0.0001 | 35 |
| ncRNA processing | <0.0001 | 33 |
| Regulation of neuron differentiation | 8.00E-03 | 27 |
| Sodium ion transport | 8.00E-03 | 14 |
| Invasive growth | 6.00E-03 | 10 |
| \* Subset of enriched pathways for H3K27M vs WT cells at day 5 post-differentiation-Cluster a | | |

## Slide 2
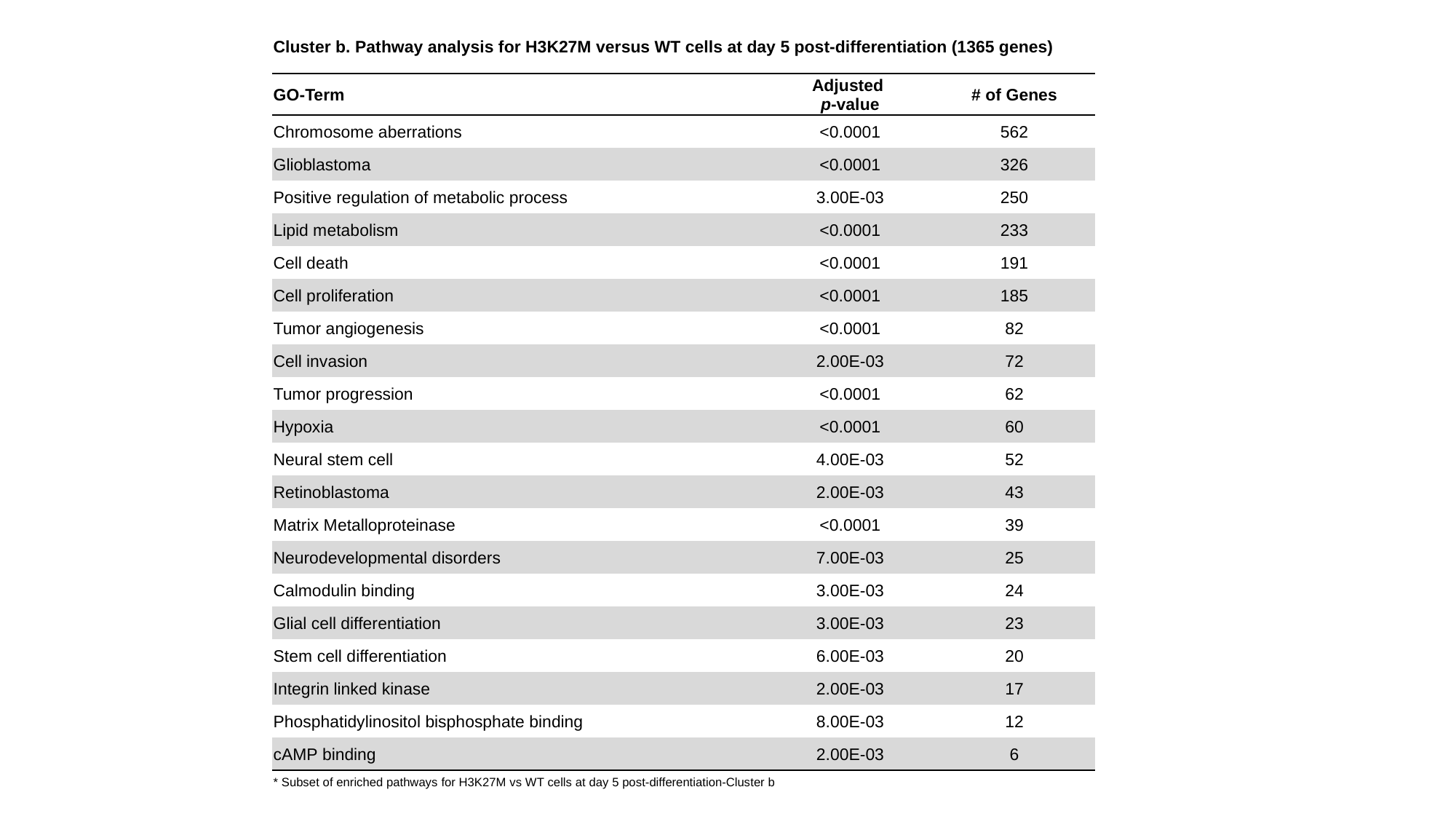

| Cluster b. Pathway analysis for H3K27M versus WT cells at day 5 post-differentiation (1365 genes) | | |
| --- | --- | --- |
| GO-Term | Adjusted p-value | # of Genes |
| Chromosome aberrations | <0.0001 | 562 |
| Glioblastoma | <0.0001 | 326 |
| Positive regulation of metabolic process | 3.00E-03 | 250 |
| Lipid metabolism | <0.0001 | 233 |
| Cell death | <0.0001 | 191 |
| Cell proliferation | <0.0001 | 185 |
| Tumor angiogenesis | <0.0001 | 82 |
| Cell invasion | 2.00E-03 | 72 |
| Tumor progression | <0.0001 | 62 |
| Hypoxia | <0.0001 | 60 |
| Neural stem cell | 4.00E-03 | 52 |
| Retinoblastoma | 2.00E-03 | 43 |
| Matrix Metalloproteinase | <0.0001 | 39 |
| Neurodevelopmental disorders | 7.00E-03 | 25 |
| Calmodulin binding | 3.00E-03 | 24 |
| Glial cell differentiation | 3.00E-03 | 23 |
| Stem cell differentiation | 6.00E-03 | 20 |
| Integrin linked kinase | 2.00E-03 | 17 |
| Phosphatidylinositol bisphosphate binding | 8.00E-03 | 12 |
| cAMP binding | 2.00E-03 | 6 |
| \* Subset of enriched pathways for H3K27M vs WT cells at day 5 post-differentiation-Cluster b | | |

## Slide 3
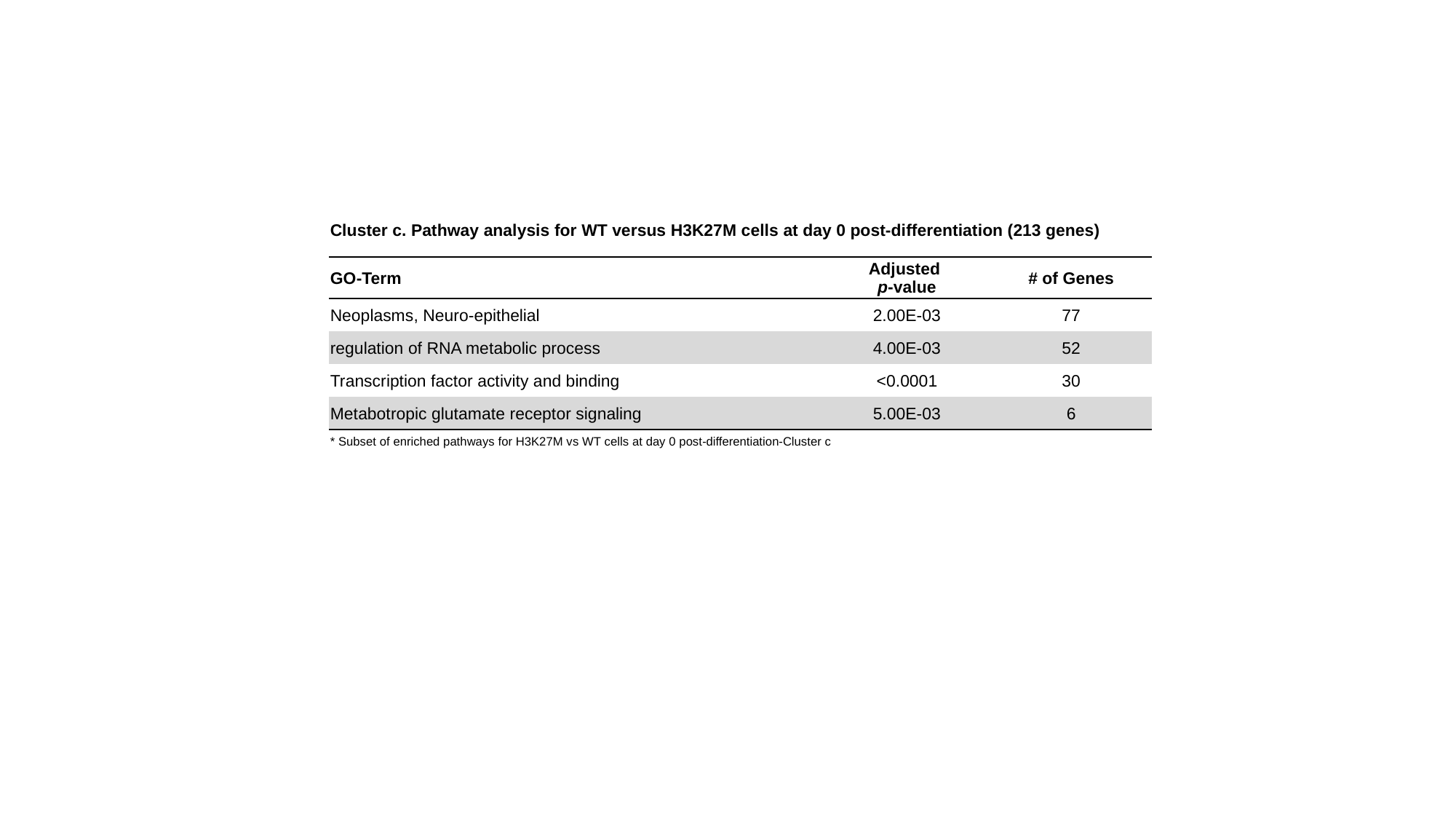

| Cluster c. Pathway analysis for WT versus H3K27M cells at day 0 post-differentiation (213 genes) | | |
| --- | --- | --- |
| GO-Term | Adjusted p-value | # of Genes |
| Neoplasms, Neuro-epithelial | 2.00E-03 | 77 |
| regulation of RNA metabolic process | 4.00E-03 | 52 |
| Transcription factor activity and binding | <0.0001 | 30 |
| Metabotropic glutamate receptor signaling | 5.00E-03 | 6 |
| \* Subset of enriched pathways for H3K27M vs WT cells at day 0 post-differentiation-Cluster c | | |

## Slide 4
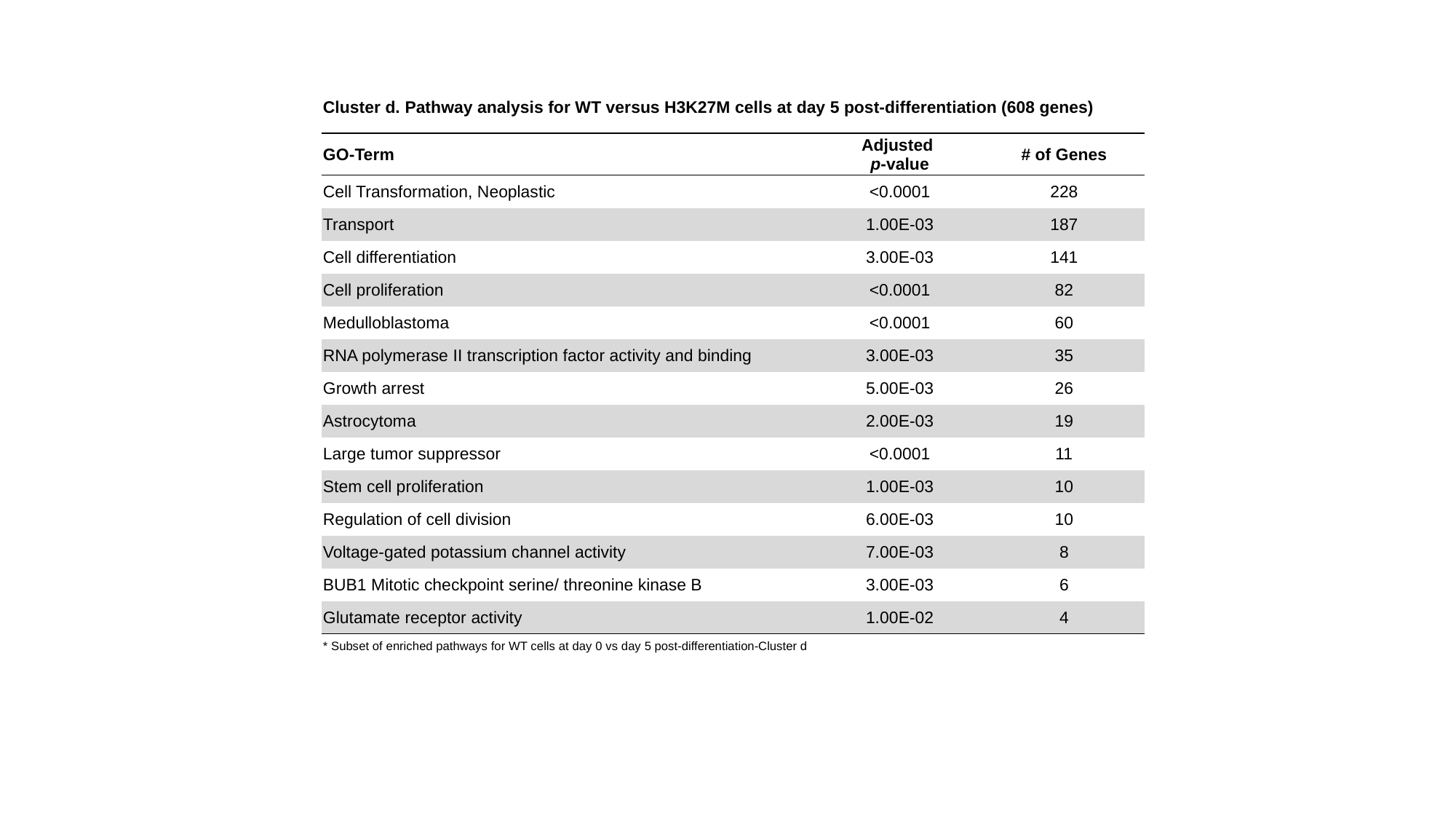

| Cluster d. Pathway analysis for WT versus H3K27M cells at day 5 post-differentiation (608 genes) | | |
| --- | --- | --- |
| GO-Term | Adjusted p-value | # of Genes |
| Cell Transformation, Neoplastic | <0.0001 | 228 |
| Transport | 1.00E-03 | 187 |
| Cell differentiation | 3.00E-03 | 141 |
| Cell proliferation | <0.0001 | 82 |
| Medulloblastoma | <0.0001 | 60 |
| RNA polymerase II transcription factor activity and binding | 3.00E-03 | 35 |
| Growth arrest | 5.00E-03 | 26 |
| Astrocytoma | 2.00E-03 | 19 |
| Large tumor suppressor | <0.0001 | 11 |
| Stem cell proliferation | 1.00E-03 | 10 |
| Regulation of cell division | 6.00E-03 | 10 |
| Voltage-gated potassium channel activity | 7.00E-03 | 8 |
| BUB1 Mitotic checkpoint serine/ threonine kinase B | 3.00E-03 | 6 |
| Glutamate receptor activity | 1.00E-02 | 4 |
| \* Subset of enriched pathways for WT cells at day 0 vs day 5 post-differentiation-Cluster d | | |

## Slide 5
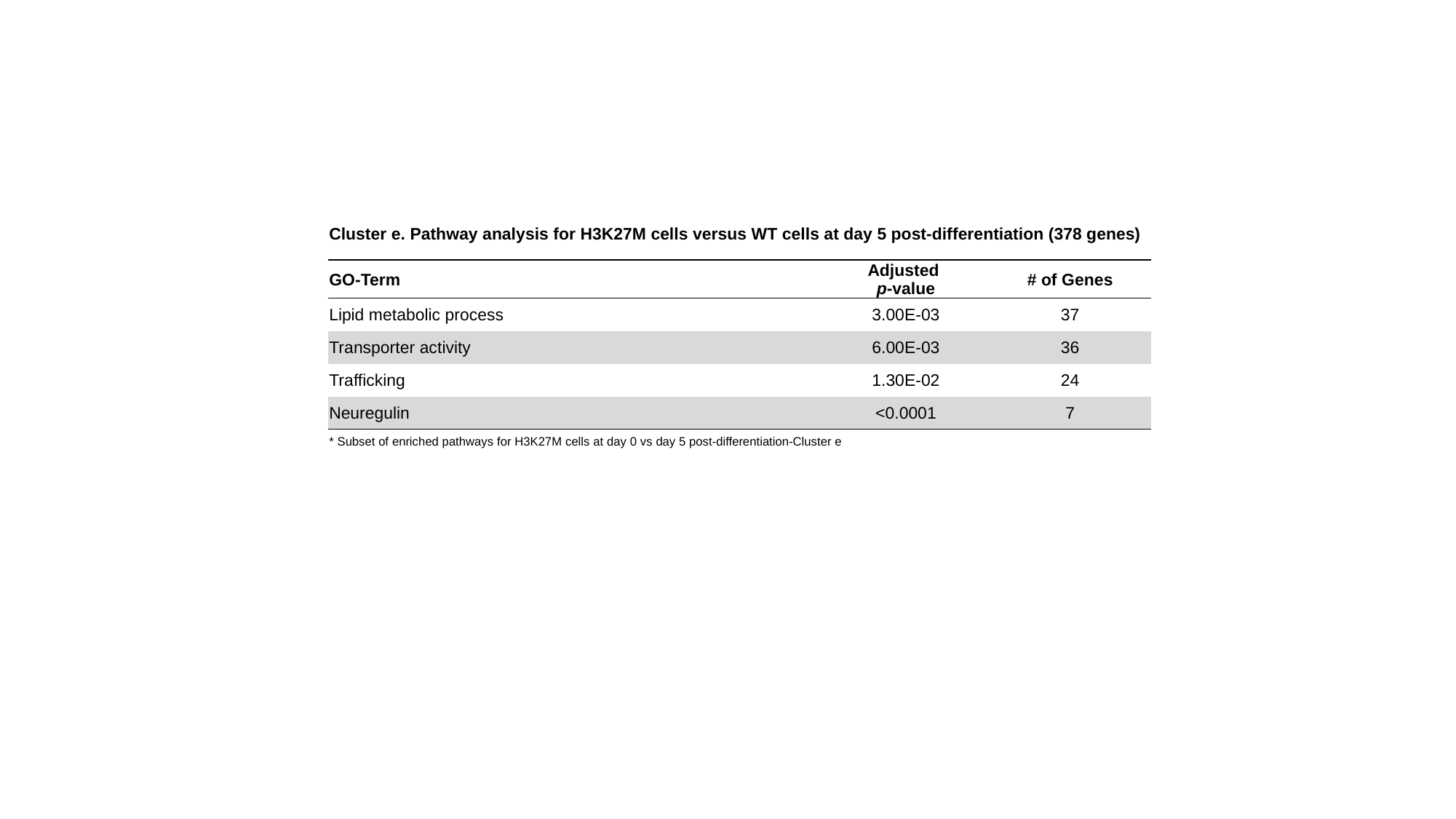

| Cluster e. Pathway analysis for H3K27M cells versus WT cells at day 5 post-differentiation (378 genes) | | |
| --- | --- | --- |
| GO-Term | Adjusted p-value | # of Genes |
| Lipid metabolic process | 3.00E-03 | 37 |
| Transporter activity | 6.00E-03 | 36 |
| Trafficking | 1.30E-02 | 24 |
| Neuregulin | <0.0001 | 7 |
| \* Subset of enriched pathways for H3K27M cells at day 0 vs day 5 post-differentiation-Cluster e | | |
